# Supplementary material for: All-optical Mie void sensor for complex nanoplastic mixtures
Source: Nat Commun. 2026 Aug 7;17:8010. doi: 10.1038/s41467-026-76199-8 (PMC13451325; doi:10.1038/s41467-026-76199-8)
Supplement: Supplementary file 1 [file 41467_2026_76199_MOESM1_ESM.pdf]

# - **Supplementary Information** -
All-optical Mie void sensor for complex nanoplastic mixtures

Dominik Ludescher[1,*], Julian Schwab[1], Serkan Arslan[1], Evelyn Kubacki[2], Monika Ubl[1], Markus Retsch[2,3], Harald. Giessen[1], and Mario Hentschel[1,*]

[1] *4th Physics Institute and Research Center SCoPE, University of Stuttgart, 70569 Stuttgart, Germany*
[2] *Department of Chemistry, Physical Chemistry I, University of Bayreuth, 95447 Bayreuth, Germany*
[3] *Bavarian Polymer Institute, Bayreuth Center for Colloids and Interfaces, Bayreuther Institut für Makromolekülforschung, and Bavarian Center for Battery Technology (BayBatt), University of Bayreuth, 95447 Bayreuth, Germany*
[*] *Corresponding authors: Mario Hentschel (m.hentschel@pi4.uni-stuttgart.de) and Dominik Ludescher (d.ludescher@pi4.uni-stuttgart.de)*

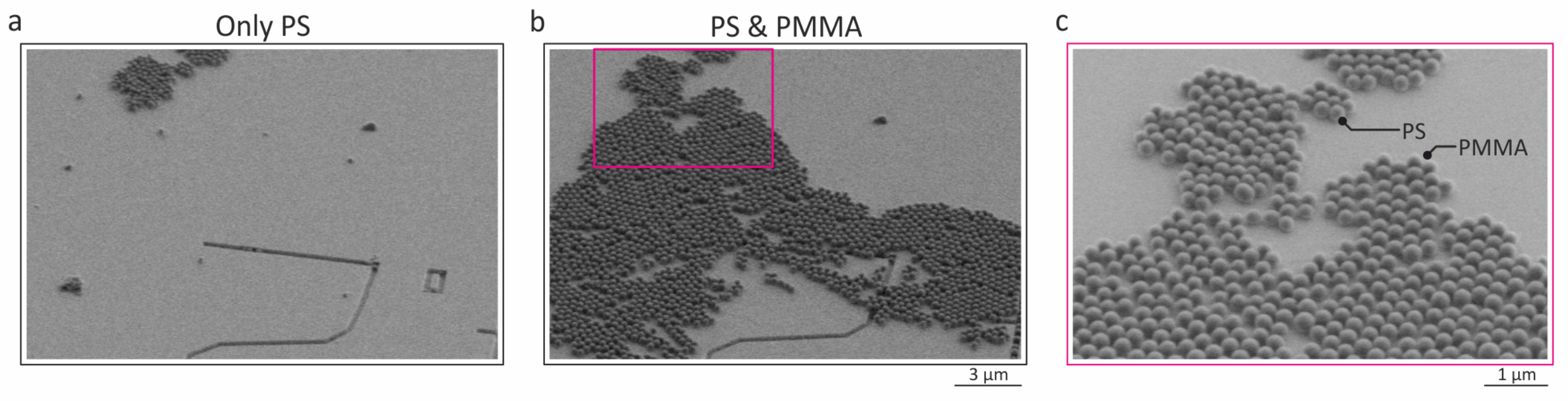

SI 1: Comparison of PS and PMMA particles. SEM images are used to compare PS and PMMA particles. **a**, SEM image only showing PS particles, forming a cluster in the top-left region. **b**, SEM image after additional deposition of PMMA particles on the same sample, without removing the initial PS particles. **c**, Magnified view of the marked pink area, where PS and PMMA particles are located adjacent to each other. No distinct optical contrast between the two particle types is observed in the SEM images.

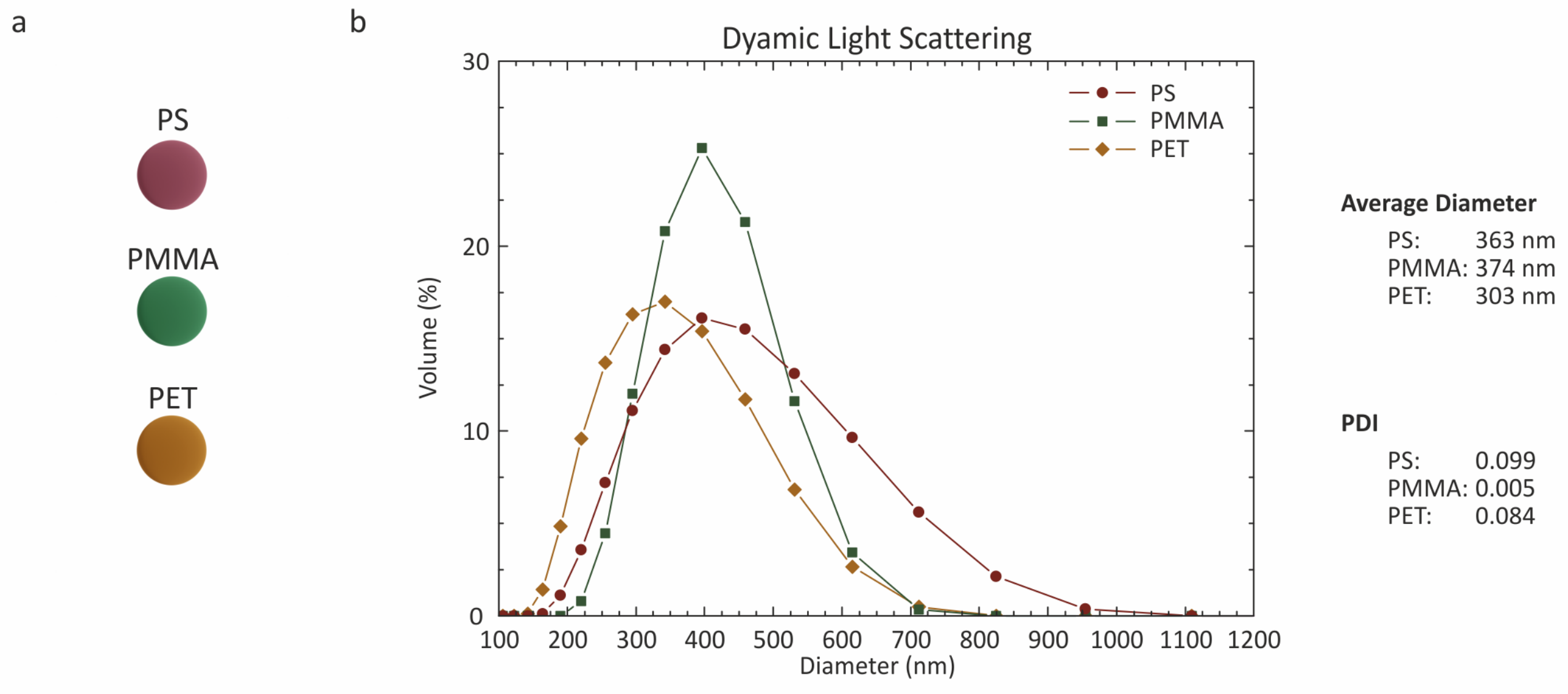

SI 2: Dynamic light scattering measurements for different particle materials. **a**, Measured particle size distributions obtained by DLS for PS, PMMA, and PET nanoparticles. **b**, Volume percentage as a function of particle diameter, with the corresponding average diameter and polydispersity index (PDI) indicated for each material.

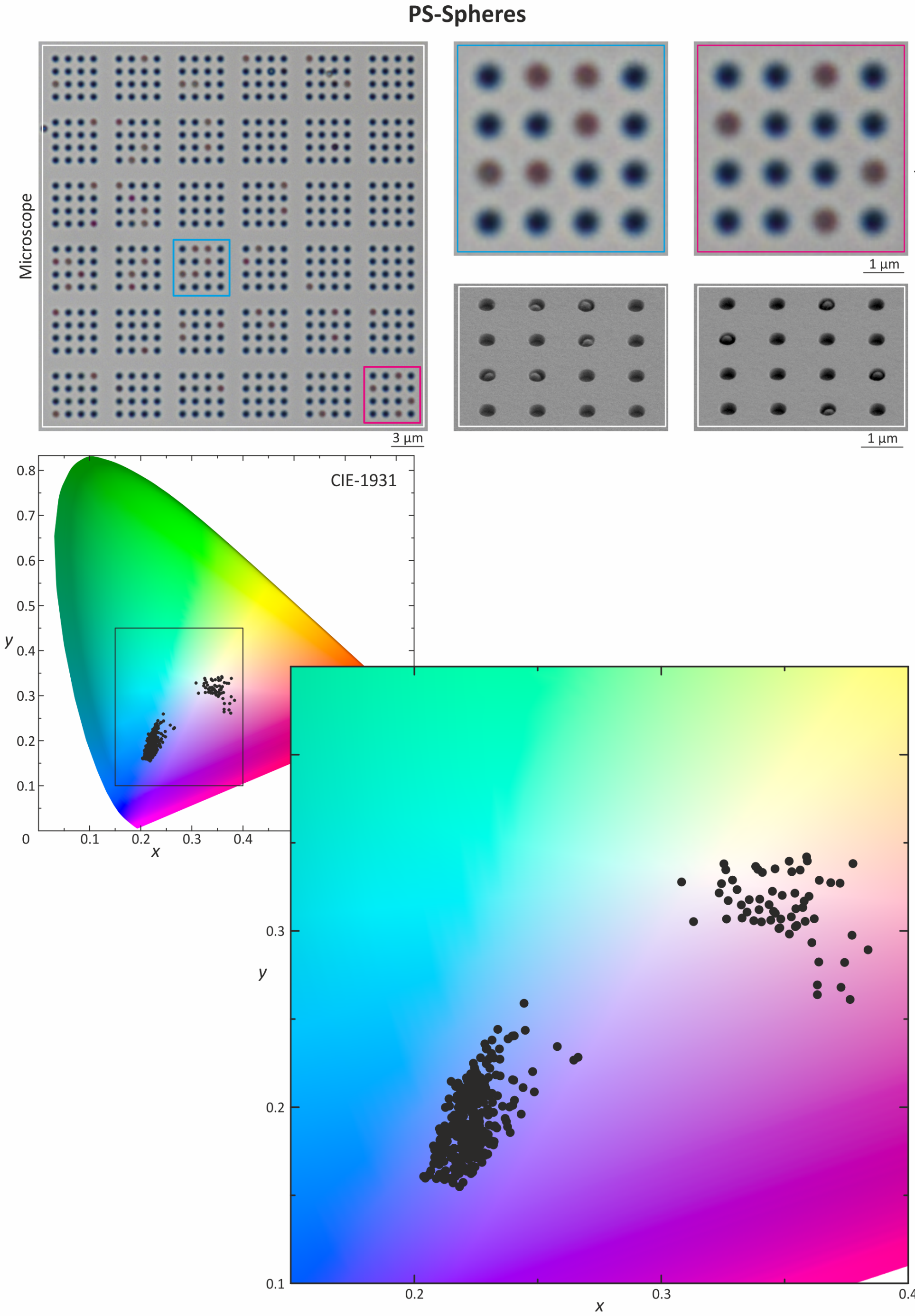

SI 3: Color reference measurement for PS particles. PS particles are deposited to determine the characteristic color of voids filled with PS. (Top) Optical image of the detection strip with two magnified regions and corresponding SEM images. (Bottom) Full CIE-1931 chromaticity diagram and a magnified view of the relevant region, indicating a red color associated with PS-filled voids.

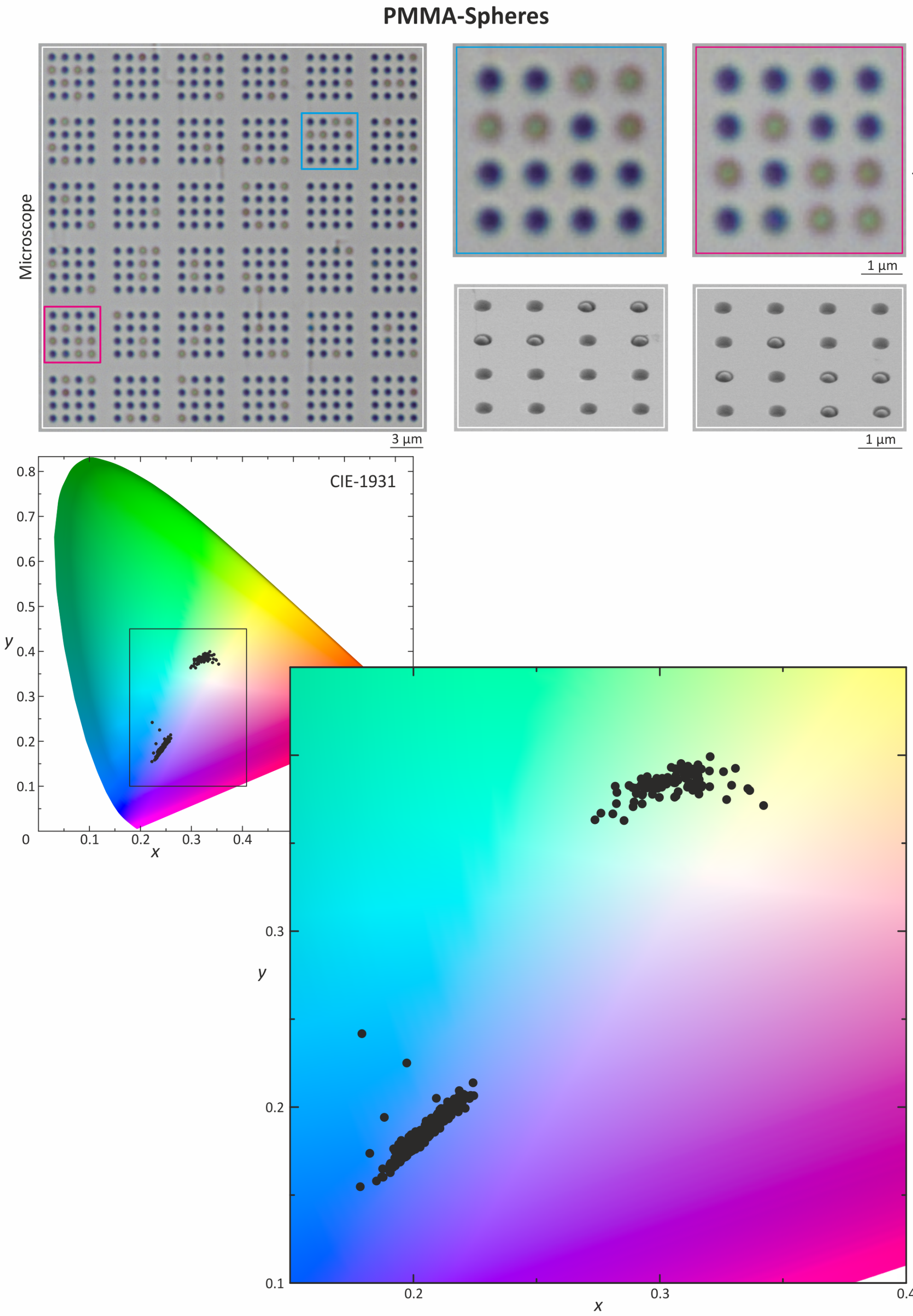

SI 4: Color reference measurement for PMMA particles. PMMA particles are deposited to determine the characteristic color of voids filled with PMMA. (Top) Optical image of the detection strip with two magnified regions and corresponding SEM images. (Bottom) Full CIE-1931 chromaticity diagram and a magnified view of the relevant region, indicating a yellow or green color associated with PMMA-filled voids.

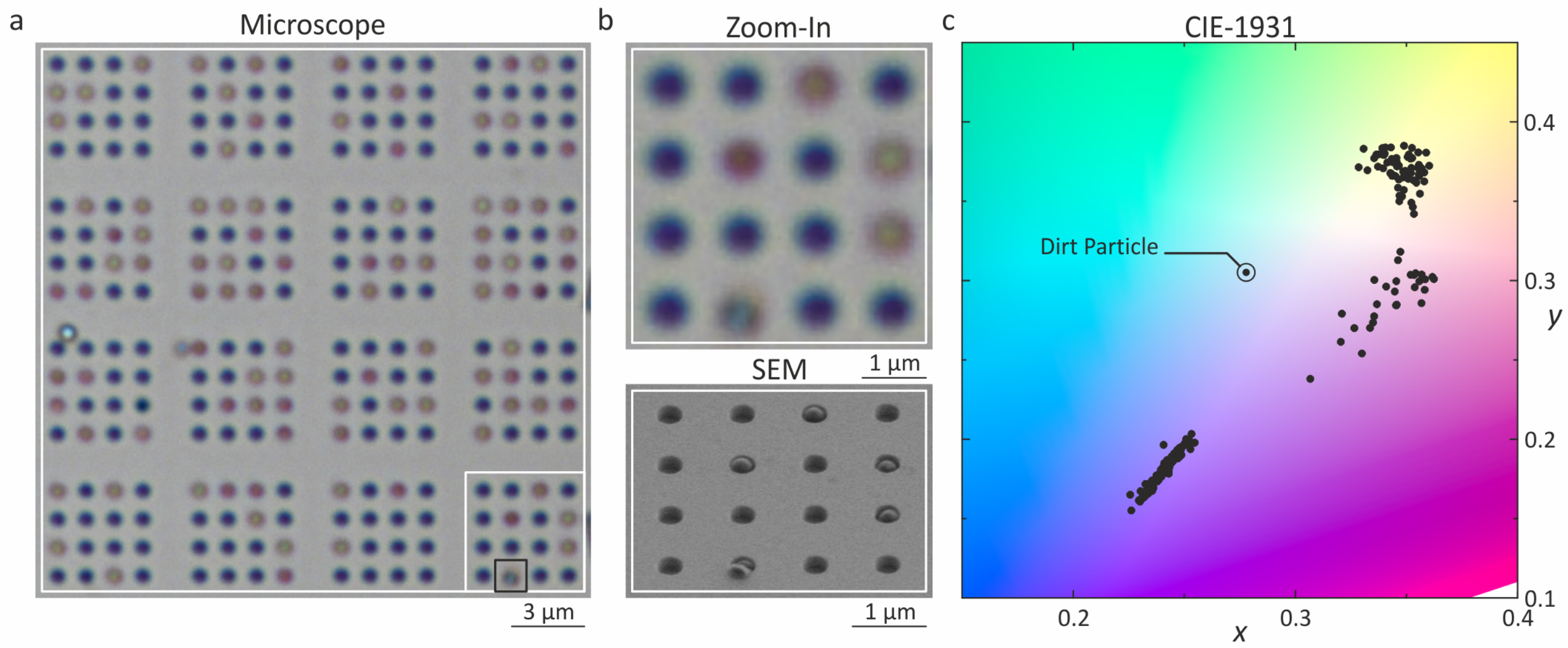

SI 5: Investigation of the influence of dirt particles. a, Microscope image of a detection strip containing a dirt particle in the lower-right corner. The void filled with the dirt particle is indicated by the black square. **b**, Magnified view of the same region with the corresponding SEM image showing the dirt particle. **c**, CIE-1931 color representation of all measured colors, revealing one clear outlier corresponding to the void filled with the dirt particle.

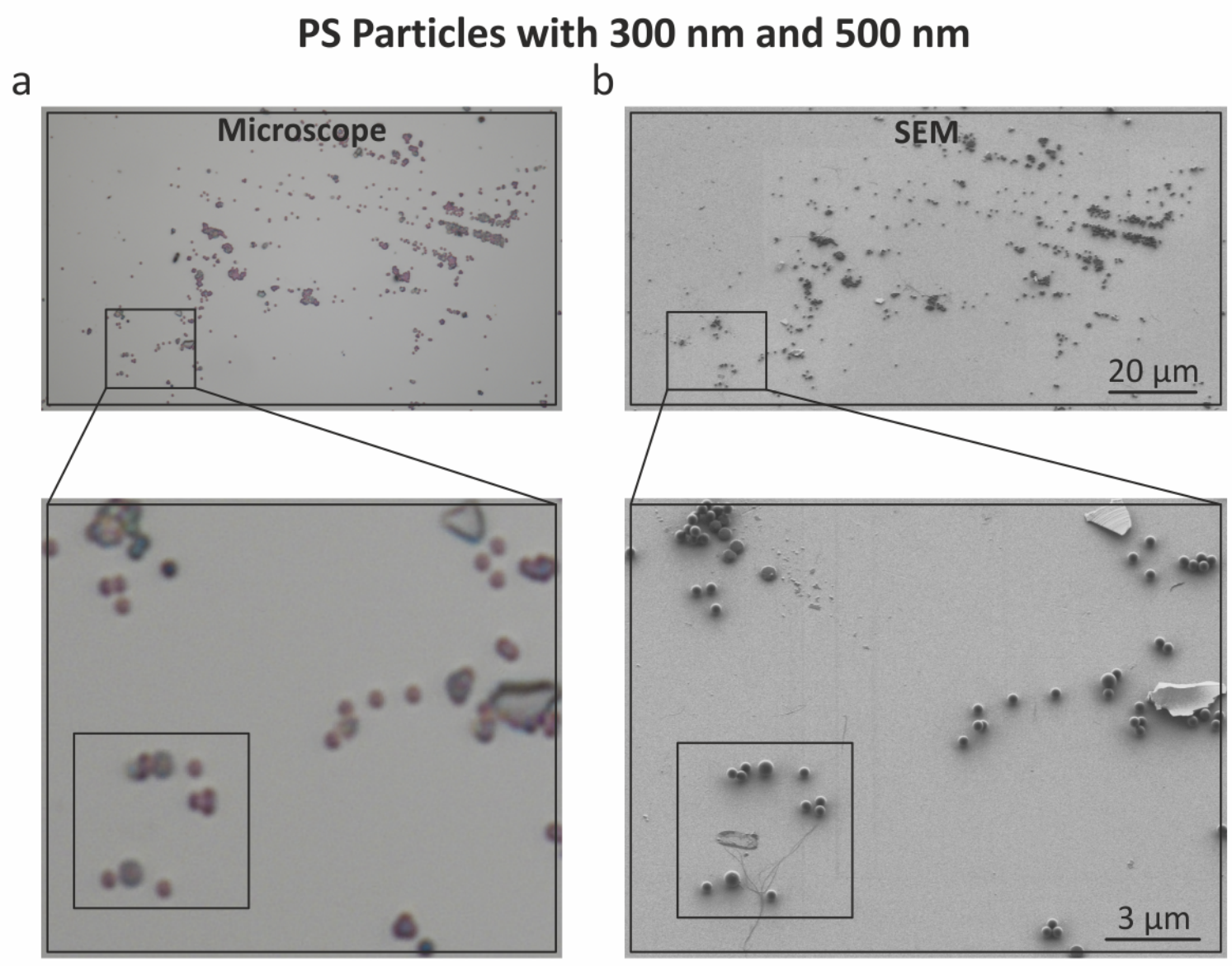

SI 6: PS particles with different diameters deposited on a flat GaAs substrate. a, Optical microscope image of PS particles with nominal diameters of 300 nm and 500 nm simultaneously deposited on a flat GaAs substrate without fabricated Mie voids. Bottom, magnified view of the area highlighted by the black box. **b**, SEM image of the same area for comparison of the particle sizes. In the optical microscope image, particle size differences and interparticle distances can only be estimated approximately.

PS & PMMA Particles with 300 nm

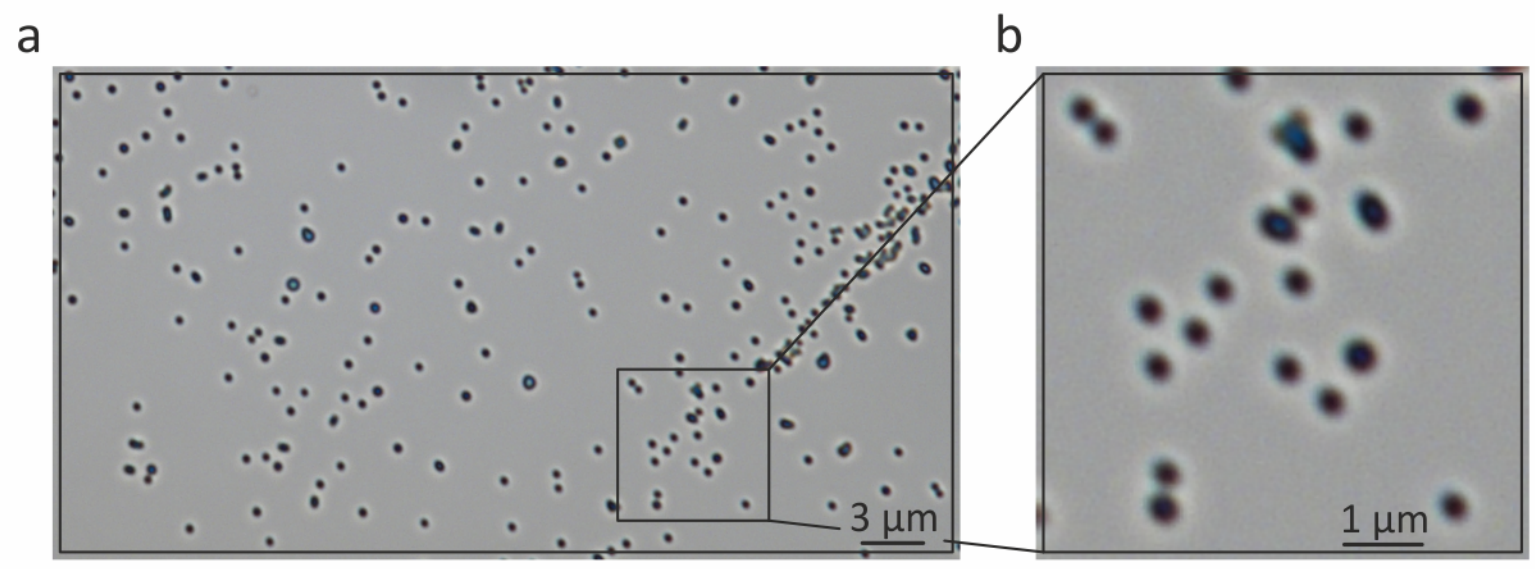

SI 7: Comparison of PS and PMMA particles on a flat GaAs substrate. **a**, Optical microscope image of PS and PMMA particles simultaneously deposited on a GaAs substrate without fabricated Mie voids. A clear distinction between the different particle types is not possible. **b**, Magnified view of the highlighted region supporting this observation.

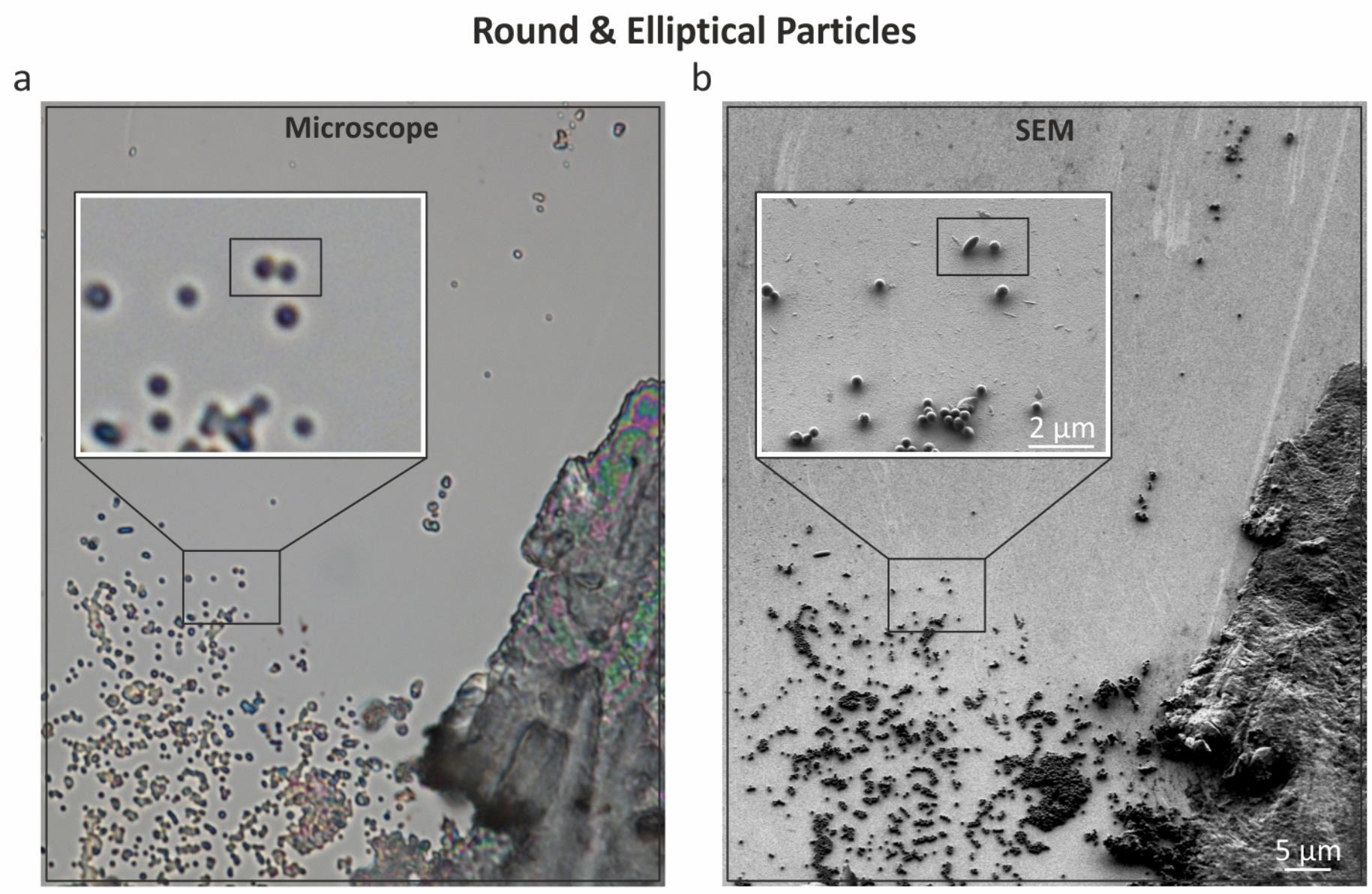

SI 8: Investigation of spherical and elliptical particles on a flat GaAs substrate. **a**, Optical microscope image of spherical and elliptical PS particles simultaneously deposited on a flat GaAs substrate without fabricated Mie void structures. The inset depicts a magnified view of the highlighted region, where no clear distinction between spherical and elliptical particles can be observed. **b**, SEM image of the same region confirming this observation. In the small inset, the black marked area contains a spherical and an elliptical particle in close proximity.

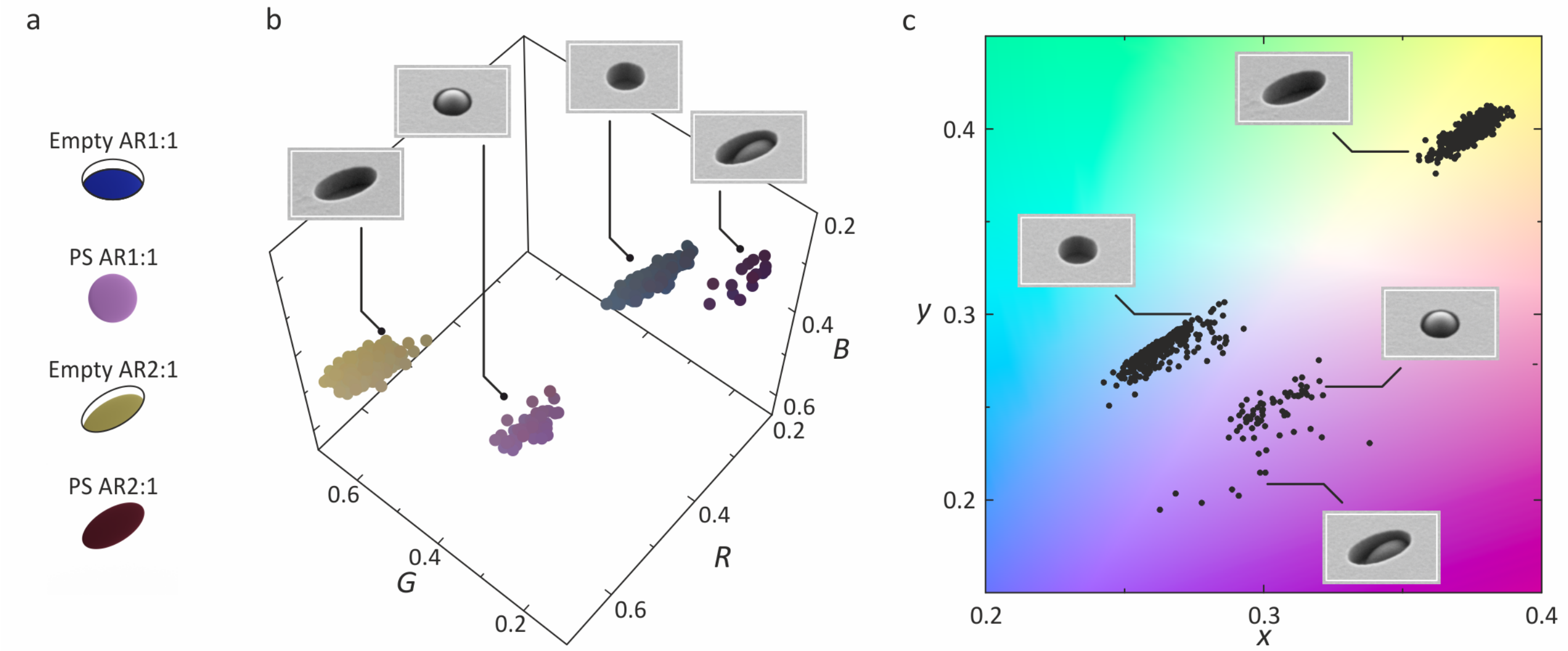

SI 9: Color space comparison. a, Four distinct void states from Figure 3 are displayed: empty and filled circular voids (blue and pink), and empty and filled elliptical voids with an aspect ratio (AR) of 2:1 (yellow and red), displayed using two different color representations. **b**, Three-dimensional RGB color space. **c**, CIE color diagram. The comparison of identical void colors across the two representations illustrates that an appropriate choice of color space enhances the separation and visibility of distinct color groups.

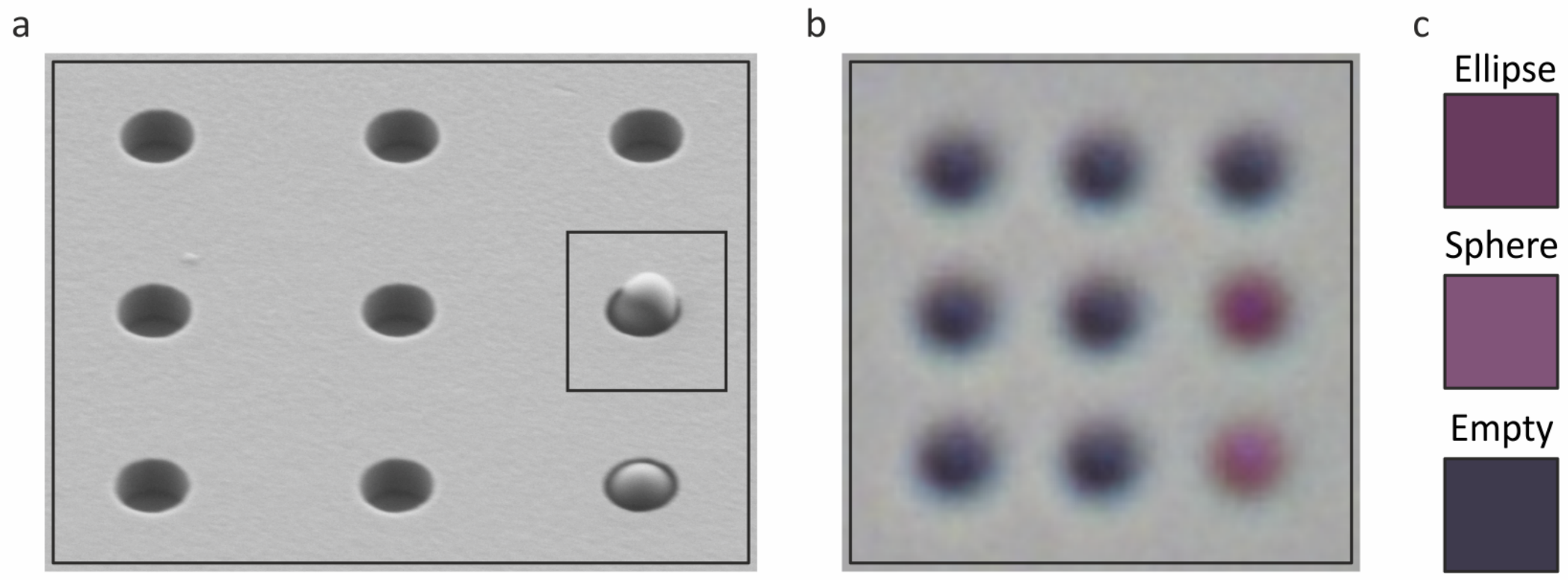

SI 10: Investigation of elliptical particles in circular voids. In rare cases, an elliptical particle can occupy a spherical void. **a**, SEM image of a 3 x 3 circular void array containing one spherical and one elliptical particle. The elliptical particle is indicated by the black square. **b**, Corresponding microscope image of the same region showing distinct colors for the two different particles. **c**, Representation of the colors corresponding to the void filled with an elliptical particle, with a spherical particle, and an empty void.

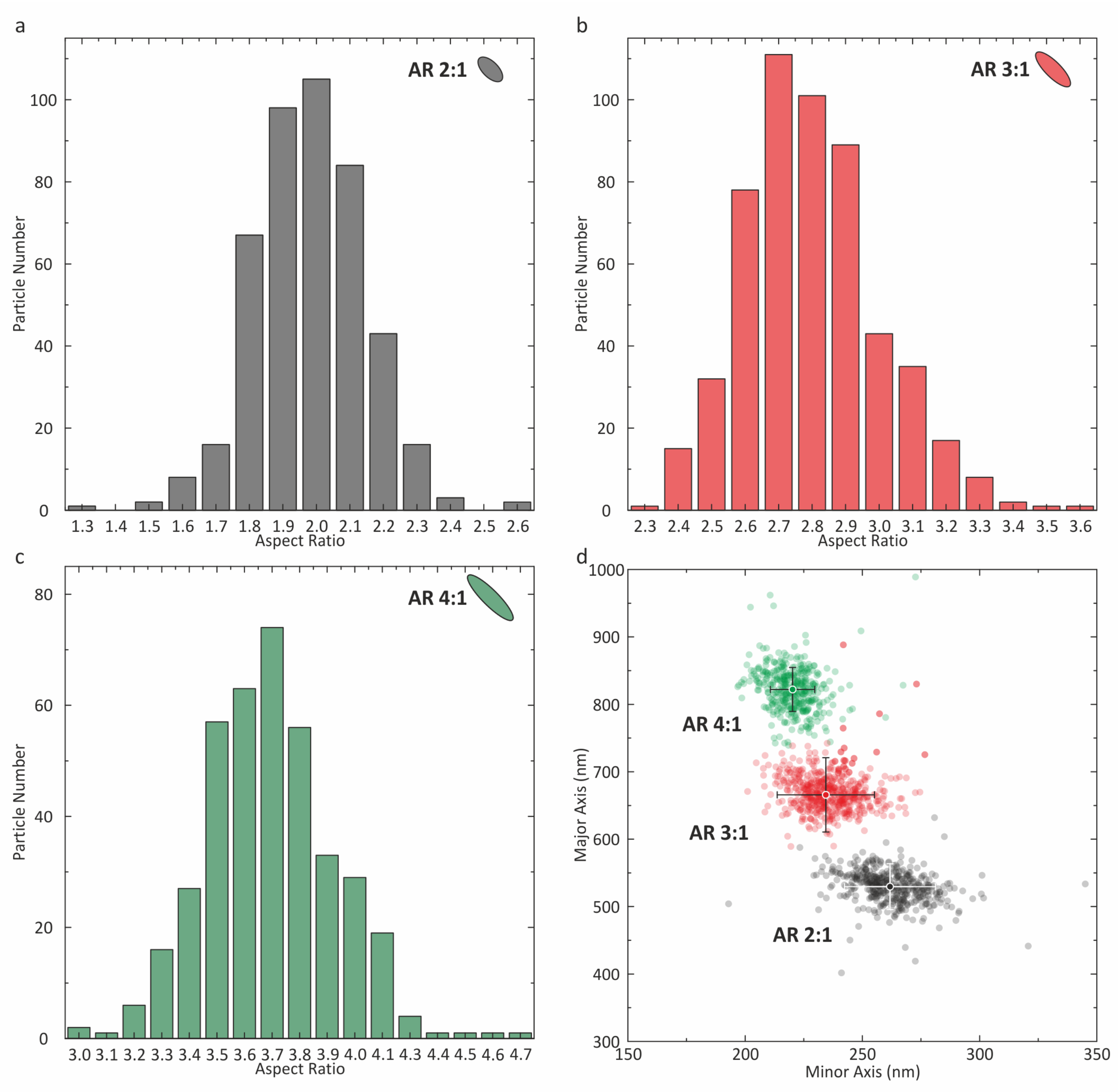

SI 11: Detailed analysis of elliptical particles. Histogram representation of the aspect ratios for particles designed with aspect ratios of **a** 2:1, **b** 3:1, and **c** 4:1. **d**, Absolute particle dimensions plotted as minor axis against the major axis for all particles (black: AR 2:1; red: AR 3:1; green: AR 4:1). The error bars represent twice the standard deviation.

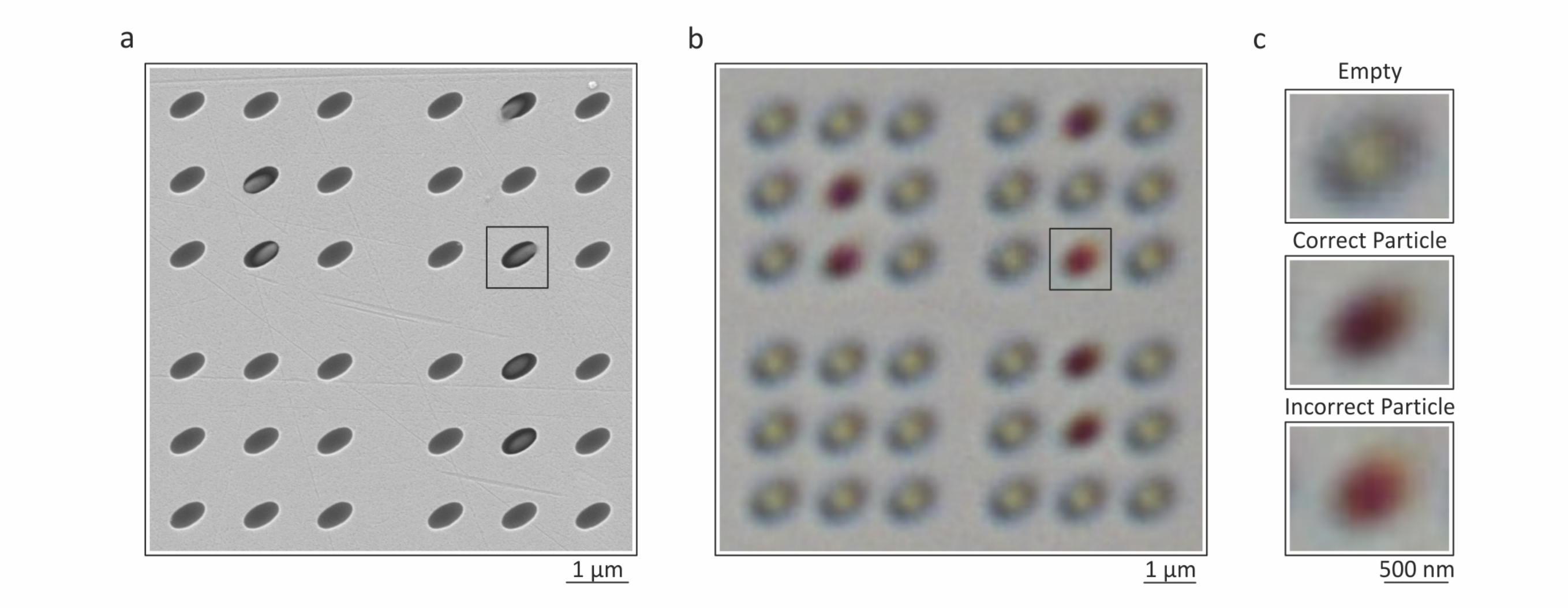

SI 12: Investigation of elongated particles with incorrect aspect ratios in elliptical voids. a, SEM image of four 3 x 3 void arrays. In the upper-right field, the indicated filled void contains an elliptical particle with an aspect ratio of 3:1, whereas all other elliptical particles exhibit an aspect ratio of 2:1. **b**, Corresponding microscope image of this area on the detection strip. The incorrectly filled void can be distinguished from the other filled voids. **c**, Magnified views on individual voids, showing an empty void, a correctly filled void, and the incorrectly filled void for comparison.

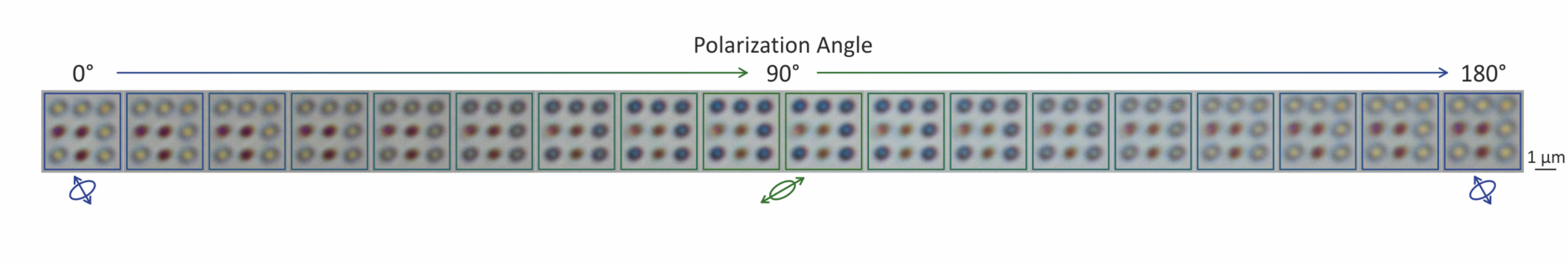

SI 13: Presentation of all polarization states. Optical microscope images used to determine the CIE distance presented in Fig. 5 are shown for all 18 different polarization states.

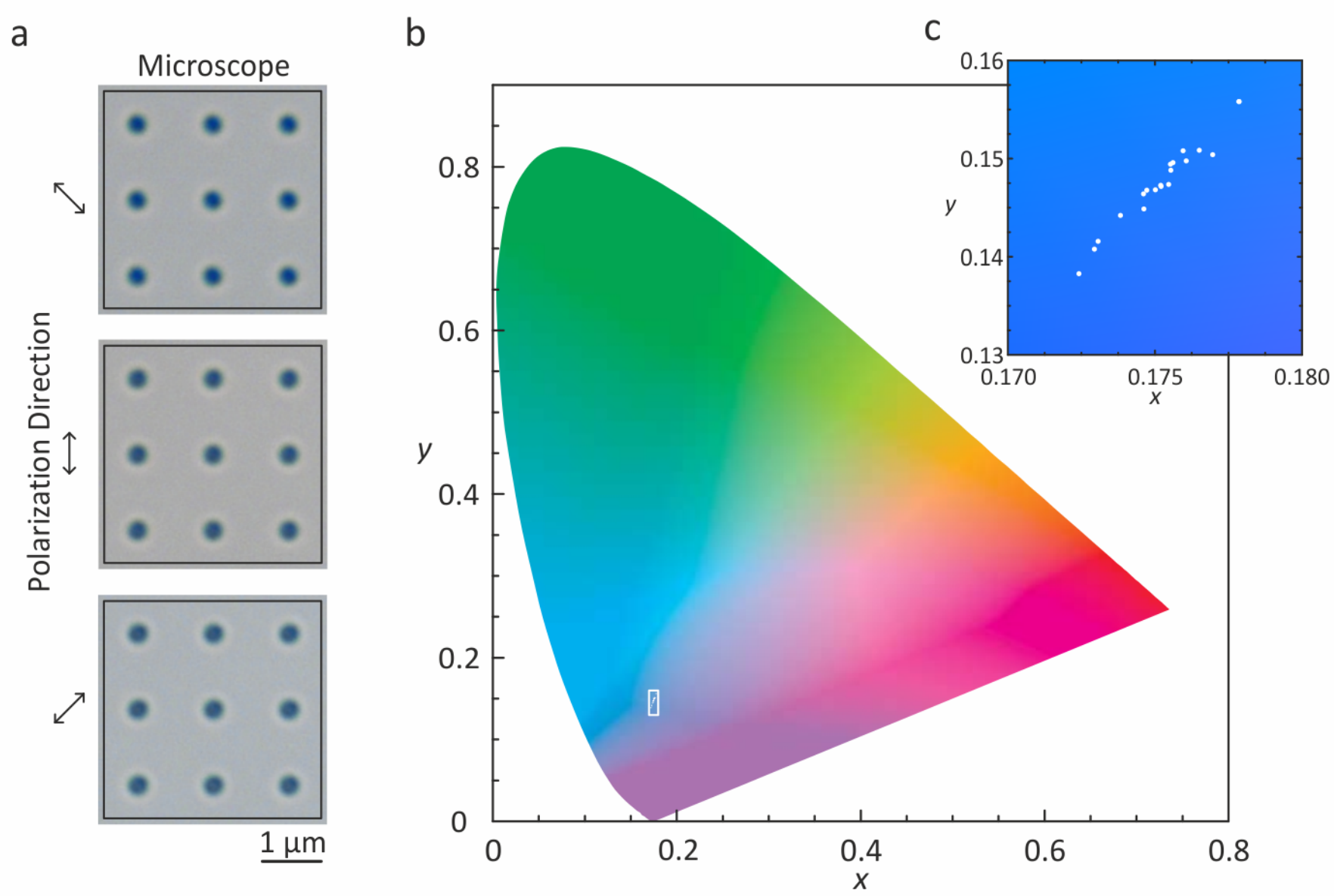

SI 14: Polarization sensitivity of empty round voids. a, Optical microscope images of nine empty round voids acquired for three different polarization states. **b**, Representation of the void colors for all polarization states in the CIE diagram, showing negligible color changes. **c**, Magnified view of the blue color range, highlighting the color stability of the round voids under polarization variation.

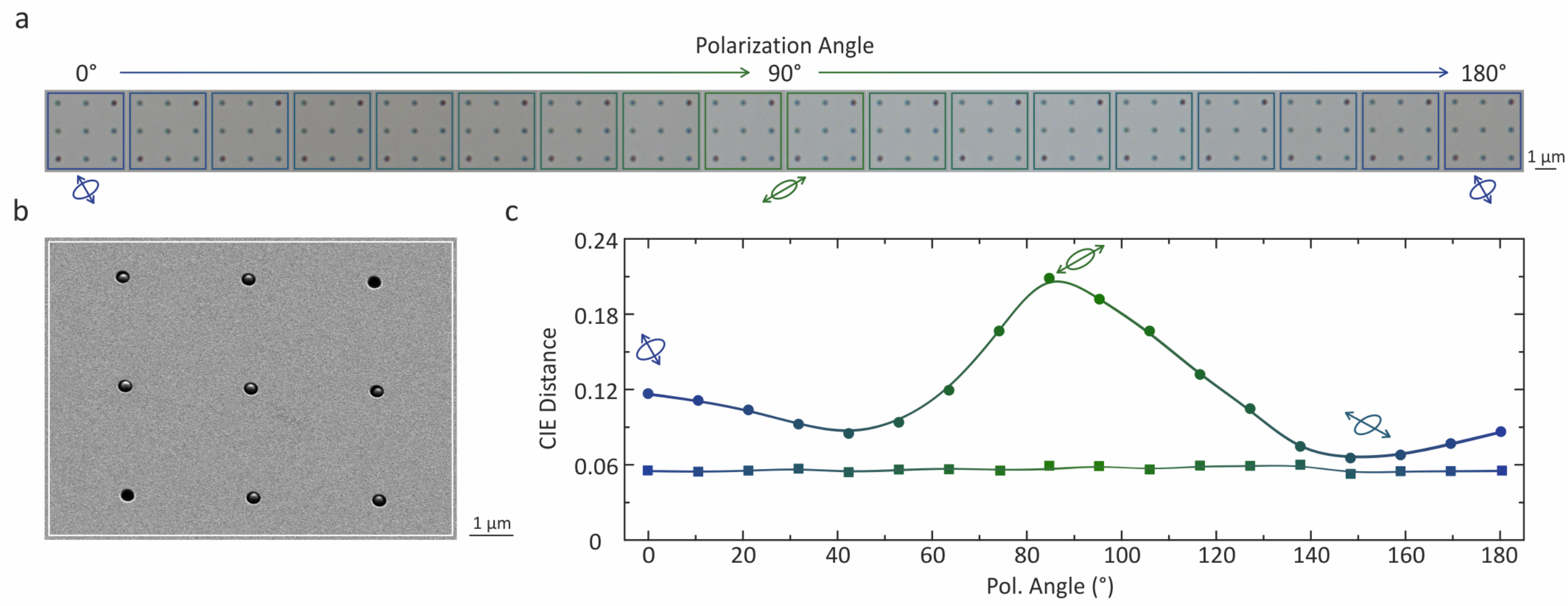

SI 15: Polarization sensitivity of filled round voids. **a**, Optical microscope images acquired for 18 different polarization states ranging from 0 ° to 180 °. **b**, SEM image of the corresponding field, indicating seven filled and two empty voids. **c**, CIE distance plotted as a function of the polarizer angle and compared with Figure 5g. Only negligible changes in the CIE distance are observed for the ideally filled round voids.

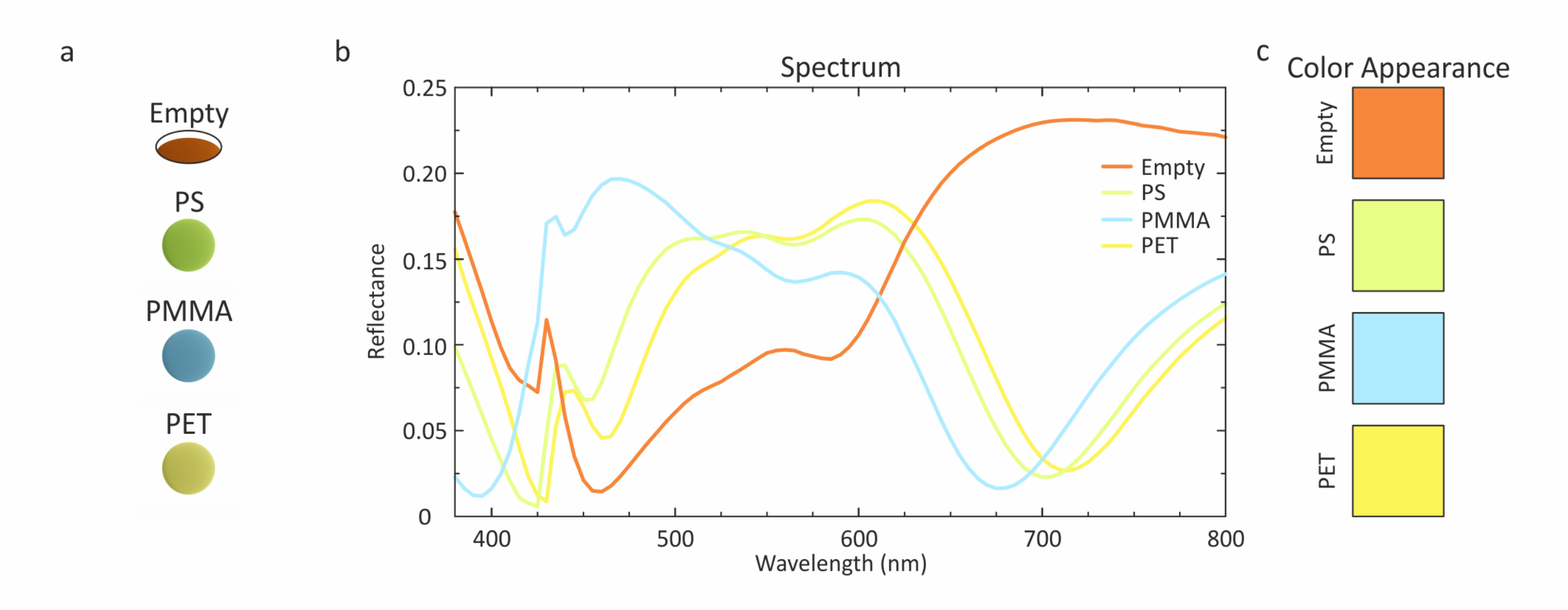

SI 16: Simulated spectra and color appearances for different particle materials. **a**, Simulations are performed for a 300 nm diameter empty void and voids filled with nanoparticles composed of PS, PMMA, and PET. **b**, Simulated reflection spectra, and **c**, Color appearance for an empty void and for a void filled with a spherical PS, PMMA, or PET particle. The void diameter is 300 nm with a depth of 200 nm.

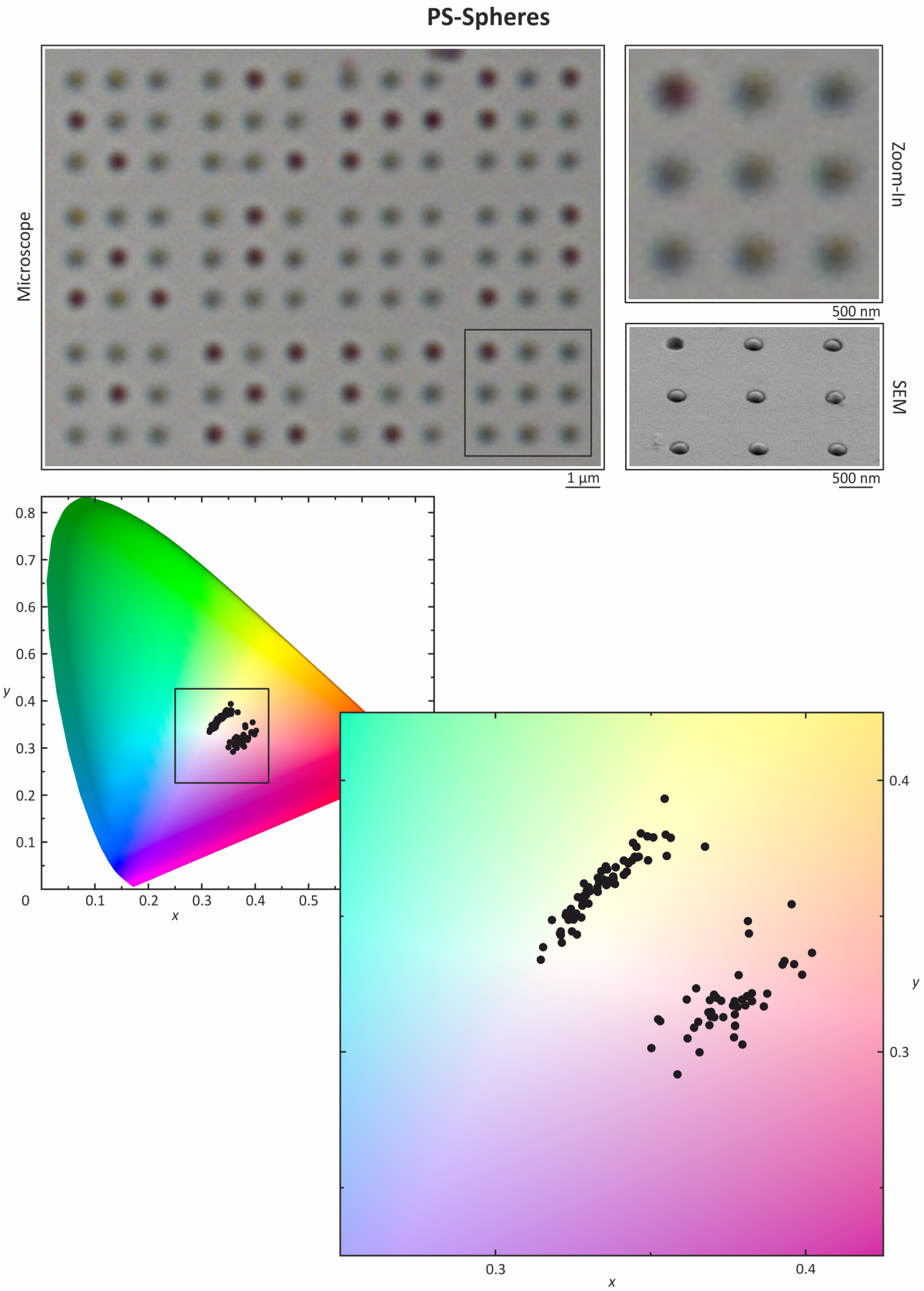

SI 17: Second color reference measurement for PS particles. PS particles are deposited to determine the characteristic color of voids filled with PS. (Top) Optical image of the detection strip with two magnified regions and corresponding SEM images. (Bottom) Full CIE-1931 chromaticity diagram and a magnified view of the relevant region, indicating a green color associated with PS-filled voids.

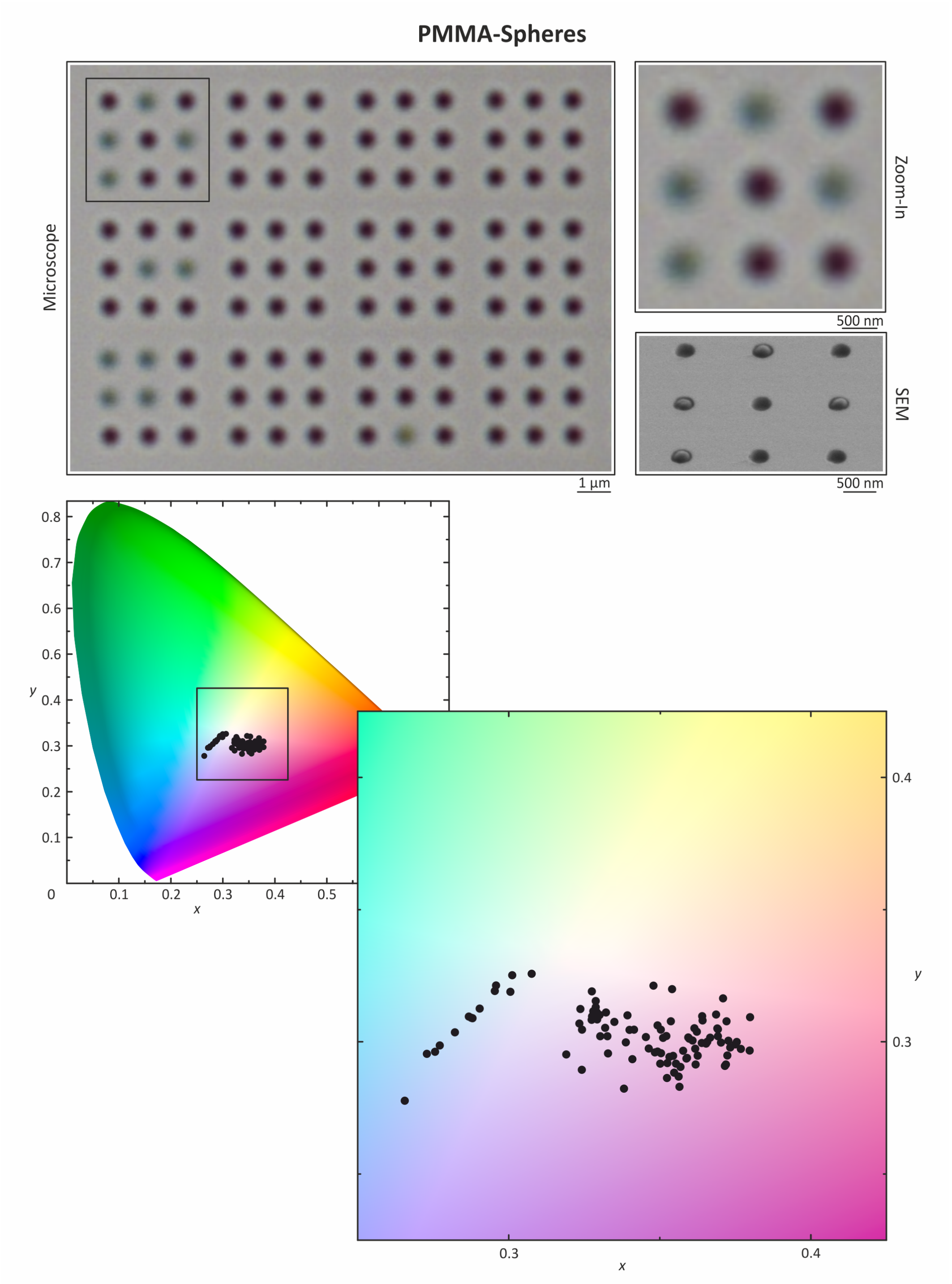

SI 18: Second color reference measurement for PMMA particles. PMMA particles are deposited to determine the characteristic color of voids filled with PMMA. (Top) Optical image of the detection strip with two magnified regions and corresponding SEM images. (Bottom) Full CIE-1931 chromaticity diagram and a magnified view of the relevant region, indicating a blue color associated with PMMA-filled voids.

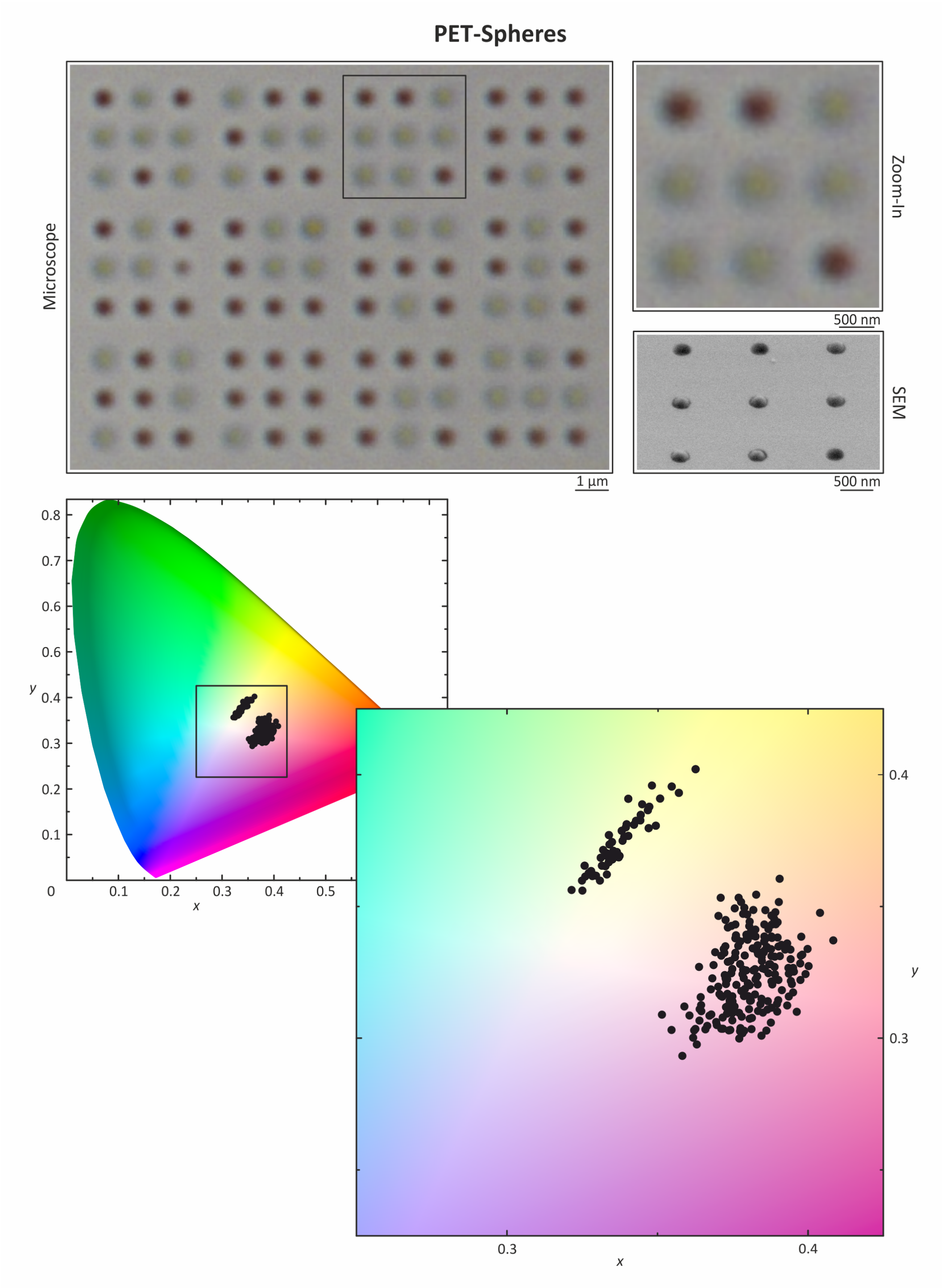

SI 19: Second color reference measurement for PET particles. PET particles are deposited to determine the characteristic color of voids filled with PET. (Top) Optical image of the detection strip with two magnified regions and corresponding SEM images. (Bottom) Full CIE-1931 chromaticity diagram and a magnified view of the relevant region, indicating a yellow color associated with PET-filled voids.
