# Supplementary material for: All-optical Mie void sensor for complex nanoplastic mixtures
Source: Nat Commun. 2026 Aug 7;17:8010. doi: 10.1038/s41467-026-76199-8 (PMC13451325; doi:10.1038/s41467-026-76199-8)
Supplement: Supplementary file 2 [file 41467_2026_76199_MOESM2_ESM.pdf]

nature portfolio

Peer Review File

All-optical Mie void sensor for complex nanoplastic mixtures

Corresponding Author: Mr Dominik Ludescher

This file contains all reviewer reports in order by version, followed by all author rebuttals in order by version.

Version 0:

Reviewer comments:

Reviewer #1

(Remarks to the Author)
The authors build upon previous work (Nature Photonics 19, 1138–1145 (2025)) and polarization-dependent Mie-type void resonators (Small, 2026, e11992) and further develop a photonic sensing platform for the detection and classification of nanoplastic particles. In the present study, arrays of nanoscale voids embedded in high-refractive-index materials are employed to enable simultaneous sensitivity to particle size, shape, and material composition. By exploiting the strong dependence of the void resonances on the local effective refractive index, different polymer materials, including PMMA, PS, and PET, can be distinguished via distinct color signatures observable under standard optical microscopy. Furthermore, by introducing circular and elliptical void geometries, the authors demonstrate selective identification of spherical and elongated particles, as well as particles with different aspect ratios. The use of polarization-resolved imaging further enhances the contrast of the optical readout.

Overall, the work represents a systematic application-oriented extension of Mie void resonator platforms, and the experimental results are clearly presented and convincing within the scope of the demonstrated model systems. However, the conceptual advance appears incremental, and the present study is not yet clearly differentiated from prior work beyond such extensions.

It is worth emphasizing that the introduction of elliptical Mie voids with varying aspect ratios enables the discrimination of particles with more complex morphologies, extending beyond simple spherical systems. This represents a meaningful step toward more realistic particle characterization, as nanoplastic particles in real environments typically exhibit diverse and irregular shapes. However, the current demonstrations remain restricted to idealized nanoparticle systems with predefined sizes and shapes, which differ significantly from the complexity encountered in real-world scenarios.

In this context, the main limitation of the current study is the lack of validation under conditions that more closely resemble realistic environments. To fully demonstrate the practical relevance and applicability of the proposed platform, it would be highly desirable to extend the experiments to more complex and less controlled systems, such as polydisperse particle distributions or samples that better approximate environmental conditions.

Therefore, I recommend major revision. The manuscript would be significantly strengthened if the authors can demonstrate the applicability of the platform in more realistic physical scenarios beyond idealized nanoparticle model systems and address the following points:

1. The manuscript acknowledges that, in rare cases, elliptical particles can occupy circular voids (see Supplementary Fig. 7), leading to mis-trapping events. However, no quantitative analysis of the frequency of such events is provided. It would be important for the authors to clarify the probability of mis-trapping and to assess whether these events could affect the overall reliability of particle classification.

Furthermore, in more complex scenarios—particularly when multiple particle sizes, shapes, and aspect ratios coexist with voids of varying geometries—the probability of such mis-trapping events may increase substantially and become non-negligible. This could potentially compromise the selectivity and reliability of the platform. A more systematic discussion or statistical evaluation of these effects would significantly strengthen the manuscript.

2. The use of polarization to enhance the color contrast between different void states is a key component of the proposed

method. In Fig. 5, the authors present measurements for three polarization configurations, including polarization aligned parallel to the elongation direction of the voids, perpendicular to it, and the unpolarized case, and use these results to identify the condition that maximizes color contrast.

However, it remains unclear whether achieving optimal discrimination requires the polarization directions to be specifically aligned with the elongation direction of the voids and the direction perpendicular to it. In particular, for elliptical voids, it would be important to clarify how sensitive the contrast enhancement is to this alignment. For example, if the polarization directions are not aligned with the elongation axis and its perpendicular direction, would the discrimination performance be significantly reduced?

Furthermore, the authors appear to assume that circular Mie voids are not polarization-sensitive, and therefore that applying the same pair of orthogonal polarization measurements (parallel and perpendicular directions) would not provide any meaningful improvement in color contrast. Could the authors verify this point experimentally or support it with a corresponding theoretical analysis? This would help clarify whether the polarization-based protocol is genuinely tied to the anisotropy of the elliptical voids.

Reviewer #2

(Remarks to the Author)
The paper reports an optical sensor for detecting plastic nanoparticles, that can differentiate between nanoplastics of different materials (PMMA, PET, PS), size, and shapes (spherical vs. elliptical shapes with different aspect ratios). The sensor is based on the concept of Mie void resonators, which are air void structures carved into a high-index GaAs substrate, that support optical resonances. The voids serve both as trapping sites for the plastic nanoparticles as well as optical readouts. The presence of nanoparticles alters the optical resonances of the Mie voids, leading to a change in color in bright-field imaging. The work provides comprehensive and convincing experimental evidence for the sensor's ability to differentiate between the size, shape, and material of plastic nanoparticles. In addition, the manuscript also leverages the polarization response of the void structures to increase the sensitivity of their sensor.

Overall, I find the manuscript interesting, well-written and timely. Mie voids are a relatively new concept in nanophotonics, where new studies are emerging increasingly frequently. The proposed sensor leverages the trapping capability and the optical resonances of Mie voids, which I find innovative. For these reasons, I feel that publication in Nature Communications would be suitable. However, I have some comments that I would like addressed first.

Major points:
1. It is clear that the voids are needed for the trapping of the particles. However, it is not obvious if the voids are needed for the optical read-out. Here, I would like to see optical control experiments, where the plastic nanoparticles are placed on e.g. flat GaAs, to clearly demonstrate that these nanoparticles cannot be differentiated without the Mie void resonances. Otherwise, if the Mie void resonances do not provide any optical functionality, then one might as well use a low-index substrate for trapping (i.e., glass) and dispel with the Mie void concept.
2. Assuming that the Mie voids are critical for optical readout, then another point that I missed in the paper are design rules. With that I mean: how are the dimensions of the voids chosen? I suppose there are design considerations for the trapping as well as for the Mie void resonances. The manuscript would benefit from being explicit on how the dimensions of the voids are chosen (and also explicitly stating these dimensions in the paper, i.e., void diameter, depth, pitch).

Minor points:
3. This is a subjective opinion, but to me, the title is not very clear. Especially the first part "size, shape, and material matter:" is bit too general. I think it could be valuable to include "Mie voids" in the title to elucidate the physical mechanism of the sensor, but I leave this decision to the authors.
4. On page 3, in the first two paragraphs of the result section, there is a short description on the trapping mechanism. In particular, the sentence on lines 106-108 prompted a few questions to me. How close do the particles have to be in size to the void dimensions? What happens if the particles are much smaller than the void dimensions, and what does "preferentially" mean in terms of success rate? I'm also wondering: Can you get a false negative result, i.e., particles that are present in the solution do not get trapped in the void and thus are not registered?
5. Along the same lines as point 4, will fabrication imperfections of the void structure impact the sensor capabilities and to what extent?
6. In relation to SI Figures 5 and 7, the authors investigate cases where unwanted particles are trapped in the voids. Without SEM imaging, how can the sensor differentiate between if such an unwanted particle is from a dirt particle or an elliptical particle tip-first?
7. In SI Figure 10, simulations of the void structure with spherical plastic nanoparticles are presented. Why are the simulated colors different from the experimental ones?
8. In SI Figure 11-13, a second set of reference measurements (i.e., pure voids without nanoplastic) are presented. Why do you need a second set of reference measurements? Are the sensors different?

Reviewer #3

(Remarks to the Author)
This manuscript demonstrated that Mie voids can selectively separate nanoplastics with different sizes and shapes by capturing them in the voids with different shapes and detect difference in materials constructing the nanoplastics by simply

observing the change in resonant color of the Mie voids filled by them with reflection optical microscope. Issue caused by nanoplastics is currently a subject of major environmental concern, which potentially impacts on lives strongly. The authors previously provided a simple and smart approach based on nanophotonics using isotropic circle-shaped Mie voids to address the issue that convenient analytical methods for nanoplastics still lack (D. Ludescher et al., Nat. Photon., 19, 1138. (2025)). The current manuscript presents the advancement from the previous work. The manuscript demonstrated the selective detection of nanoplastics with different anisotropy using Mie voids with different anisotropy, the distinction of nanoplastics composed of different materials and the advanced optical detection from the color difference under orthogonal linearly polarized light illumination to improve sensitivity. Although the basic concept of the research, optical sieve for nanoplastic detection, is the same as the author's previous work, I believe that this study is still worthy of being published in Nature communication and deserves to be widely addressed because that the current study emphasized versatility and new functionality applicable to real nanoplastics present in nature. In addition, the application of Mie voids evokes potential impacts on wide variety of scientific fields as localized surface plasmon resonance of metal nanoparticles has done.

Minor revision
Coordination x and y should be italic.

Figure 3 caption:
Regarding the description "a & b, Optical microscope and c & d, SEM images of the detection strip with elliptical (left rows) and round (right rows) voids." I believe that optical microscope images are a and c not a and b. Also I believe that SEM images are b and d not c and d.

Conclusion:
Line 312: Regarding the description "we introduce a purely optical platform that enables the simultaneous, shape…" I believe that shape detection is not purely optical.

Methods:
Descriptions should be the past tense.
Line 518: "Monodisperse PS spheres (350nm diameter) are embed-ded in an polyvinylalcohol matrix" Why "-" is between "embed" and "ded"?
Line 572: "n" of refractive index should be italic.

Version 1:

Reviewer comments:

Reviewer #1

(Remarks to the Author)
The authors have well addressed my questions. Thanks.

Reviewer #2

(Remarks to the Author)
The authors have taken great care and diligence in adressing all of the reviewers' comments, including performing and adding new experimental measurements as well as making substantial improvements to the manuscript. All of my comments have been adequately adressed, and I recommend the manuscript for publication.

Open Access This Peer Review File is licensed under a Creative Commons Attribution 4.0 International License, which permits use, sharing, adaptation, distribution and reproduction in any medium or format, as long as you give appropriate credit to the original author(s) and the source, provide a link to the Creative Commons license, and indicate if changes were made.
In cases where reviewers are anonymous, credit should be given to 'Anonymous Referee' and the source.
The images or other third party material in this Peer Review File are included in the article's Creative Commons license, unless indicated otherwise in a credit line to the material. If material is not included in the article's Creative Commons license and your intended use is not permitted by statutory regulation or exceeds the permitted use, you will need to obtain permission directly from the copyright holder.
To view a copy of this license, visit https://creativecommons.org/licenses/by/4.0/

Revision of our manuscript for Nature Communications: "Size, Shape, and Material matter: All-optical sensor for complex nanoplastic mixtures"

manuscript ID: NCOMMS-26-021878

Response to Reviewer Comments

Reviewer 1, Comment #1

> *"The authors build upon previous work (Nature Photonics 19, 1138–1145 (2025)) and polarization-dependent Mie-type void resonators (Small, 2026, e11992) and further develop a photonic sensing platform for the detection and classification of nanoplastic particles. In the present study, arrays of nanoscale voids embedded in high-refractive-index materials are employed to enable simultaneous sensitivity to particle size, shape, and material composition. By exploiting the strong dependence of the void resonances on the local effective refractive index, different polymer materials, including PMMA, PS, and PET, can be distinguished via distinct color signatures observable under standard optical microscopy. Furthermore, by introducing circular and elliptical void geometries, the authors demonstrate selective identification of spherical and elongated particles, as well as particles with different aspect ratios. The use of polarization-resolved imaging further enhances the contrast of the optical readout.*
> *Overall, the work represents a systematic application-oriented extension of Mie void resonator platforms, and the experimental results are clearly presented and convincing within the scope of the demonstrated model systems. However, the conceptual advance appears incremental, and the present study is not yet clearly differentiated from prior work beyond such extensions.*
> *It is worth emphasizing that the introduction of elliptical Mie voids with varying aspect ratios enables the discrimination of particles with more complex morphologies, extending beyond simple spherical systems. This represents a meaningful step toward more realistic particle characterization, as nanoplastic particles in real environments typically exhibit diverse and irregular shapes. However, the current demonstrations remain restricted to idealized nanoparticle systems with predefined sizes and shapes, which differ significantly from the complexity encountered in real-world scenarios.*
> *In this context, the main limitation of the current study is the lack of validation under conditions that more closely resemble realistic environments. To fully demonstrate the practical relevance and applicability of the proposed platform, it would be highly desirable to extend the experiments to more complex and less controlled systems, such as polydisperse particle distributions or samples that better approximate environmental conditions.*
> *Therefore, I recommend major revision. The manuscript would be significantly strengthened if the authors can demonstrate the applicability of the platform in more realistic physical scenarios beyond idealized nanoparticle model systems and address the following points:"*

Our Response

We thank the reviewer for their valuable comments and positive feedback. Indeed, it would be highly desirable to analyze environmental samples. However, nanoplastic research is still in its infancy and, to the best of our knowledge, well-characterized environmental nanoplastic samples are not available. The main challenge is the lack of reliable reference standards: since no established techniques exist to fully characterize such samples, a real-world sample would have an unknown nanoplastic content, if any at all. It would therefore not provide a suitable reference for evaluating our newly developed technique.

As a second-best approach, we therefore use synthetic samples designed to approximate the composition generally expected for environmental nanoplastic samples. In these synthetic samples, the nanoplastic content is known, providing a defined reference frame against which the performance of our sensor can be assessed.

Moreover, we appreciate the reviewer's recognition of our efforts to extend the existing Mie-void framework toward a more application-oriented approach. As the reviewer correctly notes, our aim is to move beyond a model based solely on spherical polymer particles and to investigate more complex polymer mixtures, including plastic particles composed of different materials (PS, PMMA, and PET) as well as particles with varying shapes.

Reviewer 1, Comment #2

> *"The manuscript acknowledges that, in rare cases, elliptical particles can occupy circular voids (see Supplementary Fig. 7), leading to mis-trapping events. However, no quantitative analysis of the frequency of such events is provided. It would be important for the authors to clarify the probability of mis-trapping and to assess whether these events could affect the overall reliability of particle classification.*
> *Furthermore, in more complex scenarios—particularly when multiple particle sizes, shapes, and aspect ratios coexist with voids of varying geometries—the probability of such mis-trapping events may increase substantially and become non-negligible. This could potentially compromise the selectivity and reliability of the platform. A more systematic discussion or statistical evaluation of these effects would significantly strengthen the manuscript."*

Our Response
We thank the reviewer for this important comment regarding the quantitative analysis of mis-trapping events. Following this suggestion, we carefully analyzed 7500 circular and 7500 elliptical voids using optical microscopy and SEM imaging to determine the probability of mis-trapping.
For spherical particles in elliptical voids, no mis-trapping events were observed, corresponding to a probability of in this case zero. For ellipsoidal particles trapped tip-first in circular voids, 11 events were identified among 7500 analyzed voids, corresponding to a mis-trapping probability of 0.15 %.

Action Taken:
We have added two paragraphs to the Experimental Results section to give a quantitative analysis of the mis-trapping probability. It now reads as follows:

1) **Experimental Results**
   Shape-sensitive detection While spherical particles are not observed to be trapped in elliptical voids in our experiment, ellipsoidal particles were trapped tip-first in circular voids in very rare cases (Supplementary Fig. 10).
2) **Experimental Results**
   Shape-sensitive detection In total, 7500 circular voids were analyzed, among which 11 contained an ellipsoidal particle trapped tip-first, corresponding to a mis-trapping probability of 0.15 %.

Reviewer 1, Comment #3

> *"The use of polarization to enhance the color contrast between different void states is a key component of the proposed method. In Fig. 5, the authors present measurements for three polarization configurations, including polarization aligned parallel to the elongation direction of the voids, perpendicular to it, and the unpolarized case, and use these results to identify the condition that maximizes color contrast.*
> *However, it remains unclear whether achieving optimal discrimination requires the polarization directions to be specifically aligned with the elongation direction of the voids and the direction perpendicular to it. In particular, for elliptical voids, it would be important to clarify how sensitive the contrast enhancement is to this alignment. For example, if the polarization directions are not aligned with the elongation axis and its perpendicular direction, would the discrimination performance be significantly reduced?"*

Our Response
We thank the reviewer for this insightful comment. It motivated additional experiments that led to additional results that deepened the understanding of the role of polarization alignment and how

ideal discrimination between different void states can be achieved. We performed two independent polarization-dependent measurements and added them to the manuscript:

1) A systematic variation of the polarizer in the illumination path, adding additional data and insight to Fig 5 (details below).
2) Measurements utilizing a crossed-polarizer geometry (illumination vs collection). These measurements allow us to spatially localize the position of a particle all-optically inside a nanoscale void.

To clarify this point, and to assess how sensitively the contrast enhancement depends on the alignment of the polarizer relative to the void direction, we systematically varied the polarizer angle from alignment along the short axis to alignment along the long axis, and back. The resulting CIE distance as a function of the polarizer angle is presented in Fig. 5g. The data exhibits the expected behavior for an ellipse as a C_2-symmetric element. As for the differential measurement, one would intuitively expect that the difference between the two main axes of the ellipse would result in the largest differential contrast. The new data demonstrates that this is not the case, as the color contrast corresponds to a distance in color space, and the colorimetric shift of the resonances under different polarization directions cannot be intuitively predicted. We believe this is an important additional insight. Optimization in contrast and thus discrimination can be easily obtained at the microscope itself without a priori knowledge.

Moreover, we have added a new figure, now Fig. 6, in which crossed polarizers are used. This configuration enables us to identify where within an individual void the particle is located by analyzing the spatial distribution of the colors. This additional experiment expands the approach from the use of single polarizers to a two-polarizer configuration.

Action Taken:

We have revised Figure 5 and added Figure 6 to the manuscript. Accordingly, we have updated the Experimental Results section titled "Advanced and polarization-dependent image analysis", revised the corresponding figure captions, and added a new Supplementary Figure. Moreover, we have adjusted the Methods section entitled "Microscopic Imaging". The revised text now reads as follows:

1) **Experimental Results**

Expansion of Figure 5 – Polarization-dependent color contrast enhancement.
We have revised Figure 5 by adding further experimental results. Specifically, we have investigated the influence of the polarization angle in greater detail by including more polarization states and plotting the CIE distance as a function of the polarization angle. Moreover, we expanded the analysis of differential imaging to provide a more detailed assessment of its impact on color-contrast enhancement.

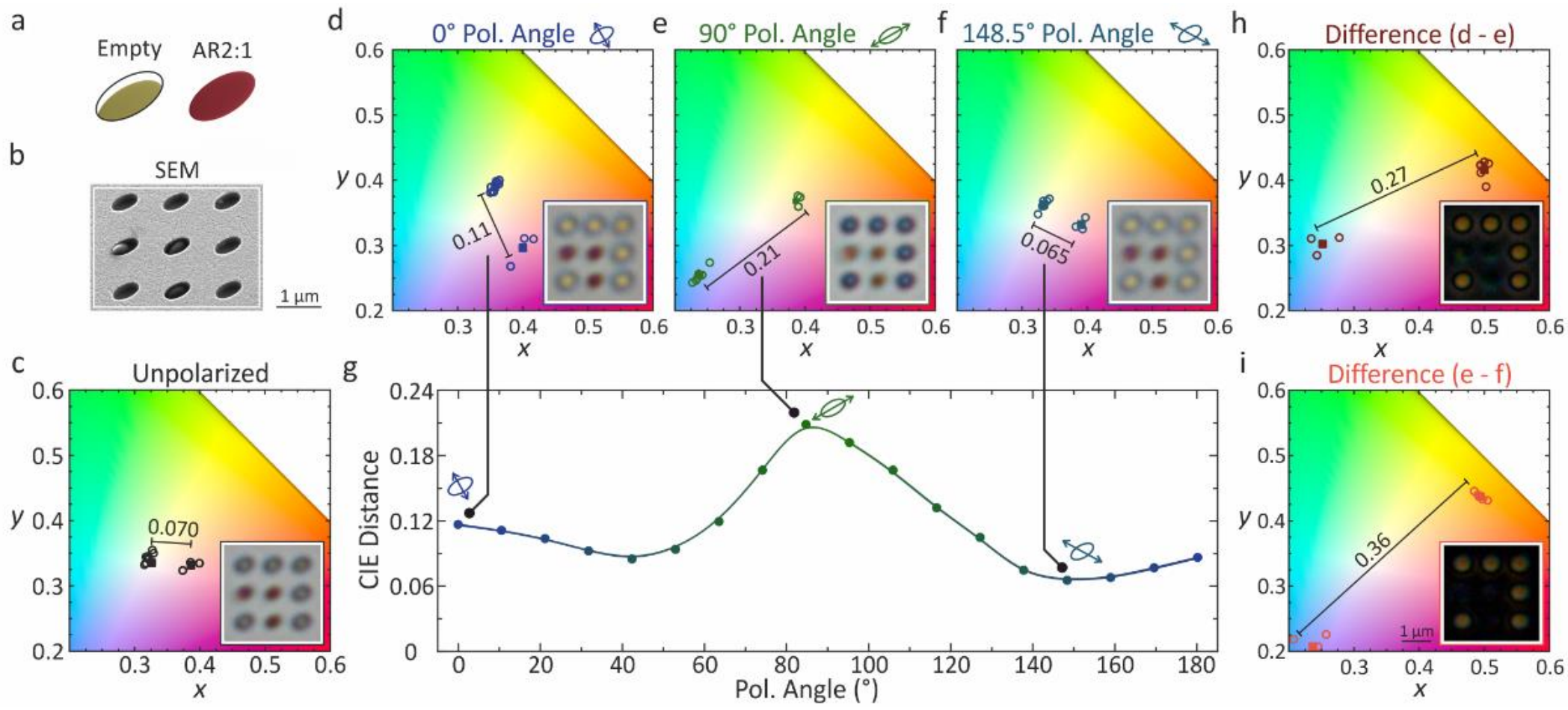

2) **Experimental Results**

Caption of Figure 5: Polarization-dependent color contrast enhancement. Detection of ellipsoidal particles is optimized through polarization-sensitive imaging and differential analysis of different polarizations. **a**, The deposited ellipsoidal particles have an aspect ratio of 2:1. **b**, SEM image of the examined detection field containing three filled voids. **c-f**, In the CIE-1931 diagram, represented by the *x* and *y* values, the color of the six empty and the three filled voids is displayed for each acquired image

(circles). The average color values (squares) reveal that the color separation depends on the polarization angle. The reported values correspond to the distance between the mean color coordinates in the CIE diagram. **g**, CIE distance as a function of polarizer angle, showing a maximum color separation for polarization aligned along the long void axis and a minimum separation at a polarization angle of 148.5 °. **h & i**, Differential images obtained from images **d** and **e**, and from images **e** and **f**, respectively. The increased color contrast demonstrates that differential imaging can enhance the color contrast between empty and filled voids. (Inset) Optical microscope images recorded with three different polarizations and the difference between the two orthogonal polarizations.

3) **Experimental Results**

Advanced and polarization-dependent image analysis As demonstrated above, the detection strip can be employed as an effective sorting element for particles with different shapes. Moreover, the ellipsoidal particles exhibit polarization-dependent behavior, enabling advanced optical analysis. In certain configurations, the intrinsic color contrast between particle states becomes weak, necessitating additional contrast enhancement. To address this limitation, we employ polarization-resolved imaging, which enables an amplification of subtle color differences by comparing different polarization states.

Figure 5 illustrates this approach for ellipsoidal particles with an AR of 2:1 deposited on the detection strip, resulting in two distinct configurations: empty and particle-filled voids (Figure 5a). A SEM image of the investigated region is shown in Figure 5b, confirming the presence of six empty and three filled voids. The voids designed for particles with an AR of 2:1 possess a major axis length of approximately 610 nm, a minor axis length of 300 nm, a depth of about 350 nm, and a center-to-center distance of 1.2 µm.

We initially compare four different imaging modalities: unpolarized illumination (Figure 5c), linear polarization aligned along the short axis (Figure 5d), along the long axis of the ellipses (Figure 5e), and with a polarization angle of 148.5 ° (Figure 5f). In addition, the graph in Figure 5g displays the determined CIE distance as a function of the polarization angle, illustrating the sensitivity of the CIE color distance to changes in polarization. The corresponding images used to calculate the polarization-angle-dependent CIE distances are provided in Supplementary Fig. 13. Representing the void colors in the two-dimensional CIE color space enables quantitative comparison through distances between color values. We define the color contrast as the Euclidean distance between two points in the CIE diagram.

Moreover, we calculate the color difference between the two perpendicular polarization states depicted in Figures 5d and 5e, as presented in Figure 5h. We further determine the color difference between the two polarization states shown in Figures 5e and 5f, as depicted in Figure 5i. For each case, the corresponding microscope image is shown as an inset, and the extracted color information is represented in the CIE chromaticity diagram. Individual measurements of the six empty and three filled voids are presented as open circles, while the respective mean values, used for distance calculations, are indicated by filled squares. Under unpolarized illumination, the color separation between empty and filled voids is modest, yielding a CIE distance of 0.070. Introducing linear polarization in the illumination path leads to a pronounced enhancement of the color contrast. For polarization aligned along the short axis, the color distance increases to 0.11 and further rises to 0.21 for alignment along the long axis. The smallest color distance of 0.065 is observed at a polarization angle of 148.5°. In all polarized cases, filled voids consistently exhibit reddish hues, while the empty voids undergo a pronounced color shift from yellow to blue, depending on the polarization orientation.

A substantially larger contrast enhancement is achieved by computing the difference image between two different linear polarization states (Figures 5h and 5i). This difference operation is performed independently for each color channel by subtracting the lower-brightness value from the higher-brightness value. As a result, regions without polarization-dependent color changes appear black, while regions with strong polarization sensitivity appear bright. In this representation, the filled voids exhibit only

a weak color response, resulting in dark tones, whereas the empty voids show a pronounced polarization-dependent color change and therefore appear in brighter orange. Notably, the background is completely black in the difference image, as it exhibits no measurable polarization dependence. Here, the difference image was calculated between the orthogonal polarization states, resulting in a color contrast value of 0.27 (Figure 5h). Although this comparison might initially be expected to yield the largest color distance, an even higher value of 0.36 (Figure 5i) is obtained when calculating the difference between the polarization states corresponding to the highest and lowest CIE distances in Figure 5g. The reason for this is that the color contrast is defined as the distance in the CIE diagram, and colorimetric shifts of the resonances induced by polarization variations cannot be predicted intuitively.

Consequently, this simple analysis significantly amplifies small intrinsic color contrasts and enables a clear discrimination between particle-filled and empty voids. Polarization-dependent difference imaging thus provides an efficient strategy to enhance contrast in optical sorting and detection schemes.

4) **Supplementary Information**

Supplementary Fig. SI13: Supplementary Fig. 13 added, presenting all optical microscope images used to determine the CIE distance in Fig. 5g.

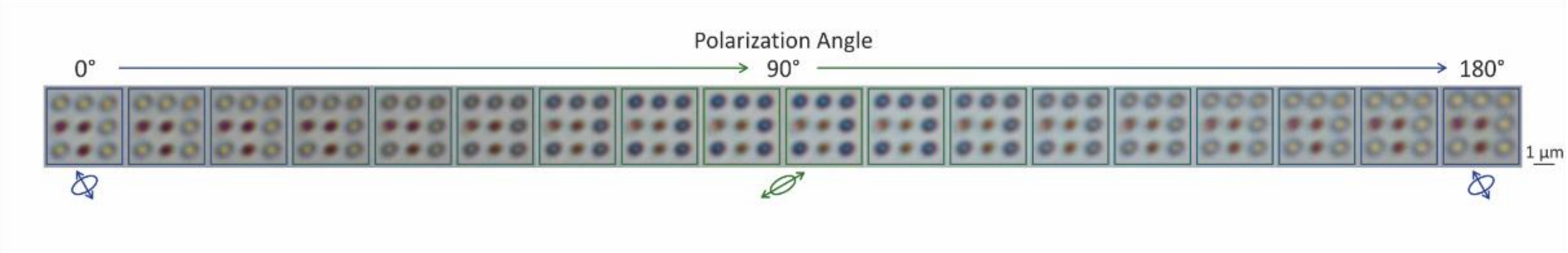

5) **Supplementary Information**

Caption of Fig. SI13: Presentation of all polarization states. Optical microscope images used to determine the CIE distance presented in Fig. 5 are shown for all 18 different polarization states.

6) **Experimental Results**

Advanced and polarization-dependent image analysis The corresponding images used to calculate the polarization-angle-dependent CIE distances are provided in Supplementary Fig. 13.

7) **Experimental Results**

Addition of Figure 6 - Crossed-polarizer analysis.

We have added an additional Figure that extends the polarization-dependent image analysis by employing two crossed linear polarizers. This approach enables the determination of particle positions within individual voids.

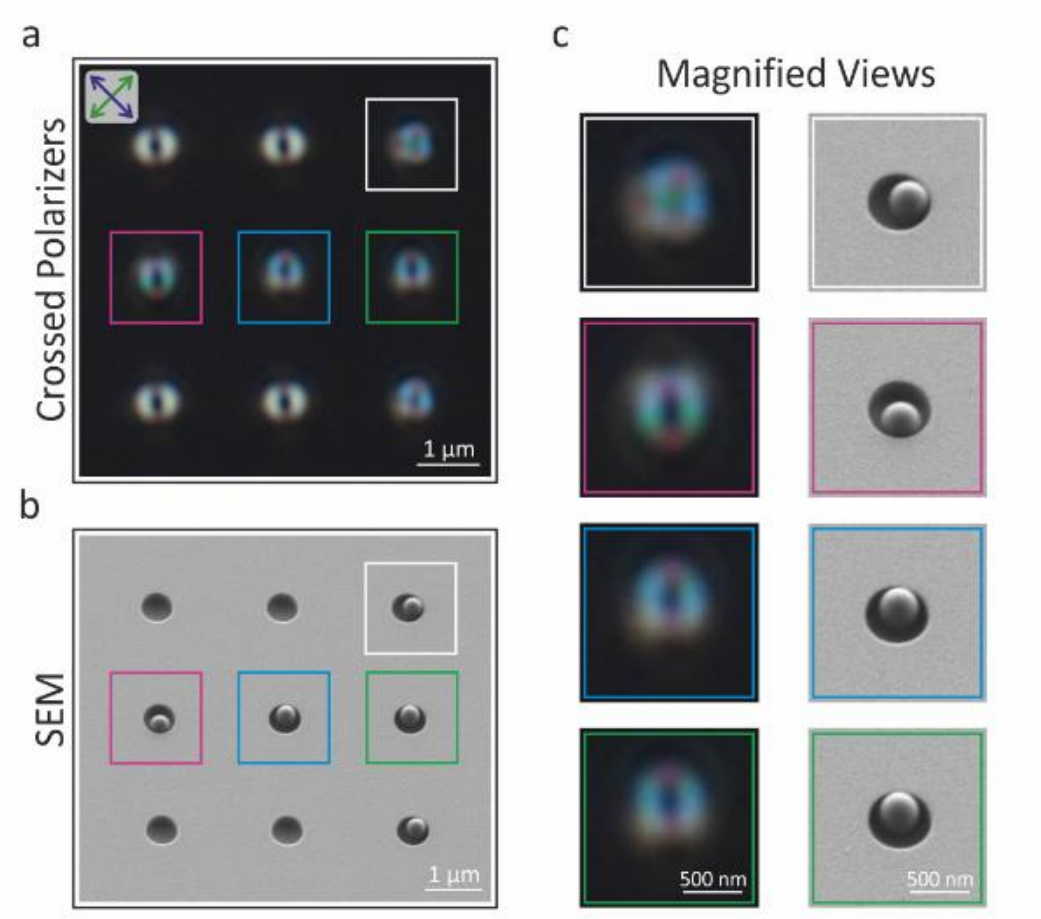

8) **Experimental Results**

Caption of Figure 6: Crossed-polarizer analysis. Particle positions within individual voids are analyzed using two crossed linear polarizers. **a**, Optical microscope image acquired under crossed-polarizer illumination. **b**, Corresponding SEM image indicating the different particle positions within the voids. **c**, Magnified views of four distinct voids.

The colored frames indicate the corresponding voids in the optical and SEM image. Comparison of the voids marked by the white, pink, and blue frames shows that the optical microscope image already provides information about the particle position. In addition, no clear difference is observed between the voids marked by the blue and green frames due to the same particle position within the void.

9) **Experimental Results**

Advanced and polarization-dependent image analysis In addition, we extended the polarization-dependent image analysis from a single linear polarizer to two linear polarizers in a crossed configuration. Figure 6a displays an optical microscope image acquired using crossed polarizers, with the corresponding SEM image shown in Figure 6b. Comparison of the optical and SEM images reveals that the particles occupy different positions within the individual voids. The voids have a diameter of approximately 560 nm and a depth of about 200 nm. In this experiment, the void dimensions are deliberately mismatched with respect to the deposited particles, which have a nominal diameter of 300 nm, to allow different particle positions within the voids. Although such states would typically be removed during the cleaning procedure, careful cleaning using low ultrasonication power made it possible to preserve them. Comparing the magnified views depicted in Figure 6c, the different particle positions within the voids can already be inferred from the spatial color distribution in the optical microscope image. The colored frames indicate the corresponding voids in the full optical microscope image. Consistently, the blue- and green-framed images show particles located at nearly the same position within the voids, resulting in a similar spatial color distribution in the optical microscope images.

10) **Methods**

Microscopic Imaging For polarization-dependent imaging with a single polarizer (Figures 3, 4, and 5), a rotatable linear polarizer is inserted into the illumination path. For crossed-polarizer imaging, a second, fixed linear polarizer is additionally inserted into the detection path (Figure 6).

<u>Reviewer 1, Comment #4</u>

“Furthermore, the authors appear to assume that circular Mie voids are not polarization-sensitive, and therefore that applying the same pair of orthogonal polarization measurements (parallel and perpendicular directions) would not provide any meaningful improvement in color contrast. Could the authors verify this point experimentally or support it with a corresponding theoretical analysis? This would help clarify whether the polarization-based protocol is genuinely tied to the anisotropy of the elliptical voids.”

Our Response

We thank the reviewer for this interesting comment. We verified the color stability under different polarizations of circular voids by analyzing both empty round voids (Supplementary Fig. 14) and the CIE color distance of filled to empty round voids in comparison to the plot given in Figure 5g (Supplementary Fig. 15). These results indicate that circular voids exhibit negligible polarization-dependent color changes. Thus, an intrinsic anisotropy of the void geometry is required for the polarizer-based protocol to enhance the color contrast effectively.

Action Taken:

We added two Supplementary Figures (SI14 and SI15) to provide further details on the polarization sensitivity of circular voids. The Experimental Results sections have been revised accordingly.

1) **Supplementary Information**

Added Supplementary Fig. SI14: To clarify the polarization sensitivity of empty round voids, this Supplementary Figure was added. Here, the color variation depending on the polarization state is depicted.

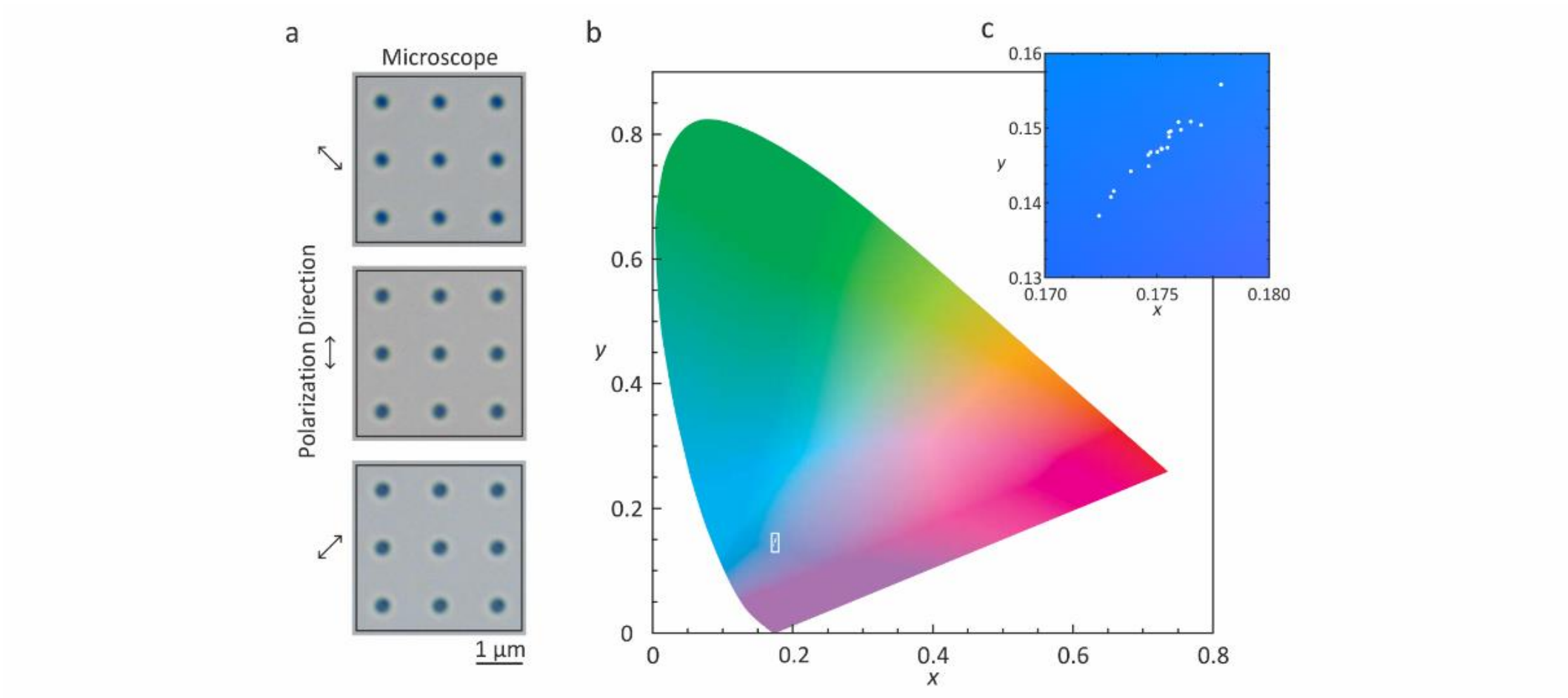

2) **Supplementary Information**

Caption of Supplementary Fig. SI14: Polarization sensitivity of empty round voids. a, Optical microscope images of nine empty round voids acquired for three different polarization states. **b**, Representation of the void colors for all polarization states in the CIE diagram, showing negligible color changes. **c**, Magnified view of the blue color range, highlighting the color stability of the round voids under polarization variation.

3) **Experimental Results**

Advanced and polarization-dependent image analysis A detailed analysis of the polarization sensitivity of round, empty voids is provided in Supplementary Fig. 14. This analysis implies that round voids exhibit no polarization sensitivity.

4) **Supplementary Information**

Added Supplementary Fig. SI15: Polarization sensitivity for filled round voids. To clarify whether the utilized polarizer-dependent protocol can also be used for round voids, the examination presented in Figure 5g was repeated for round voids filled with spherical particles. Here, no CIE color distance variation can be observed for a change of polarizer angle.

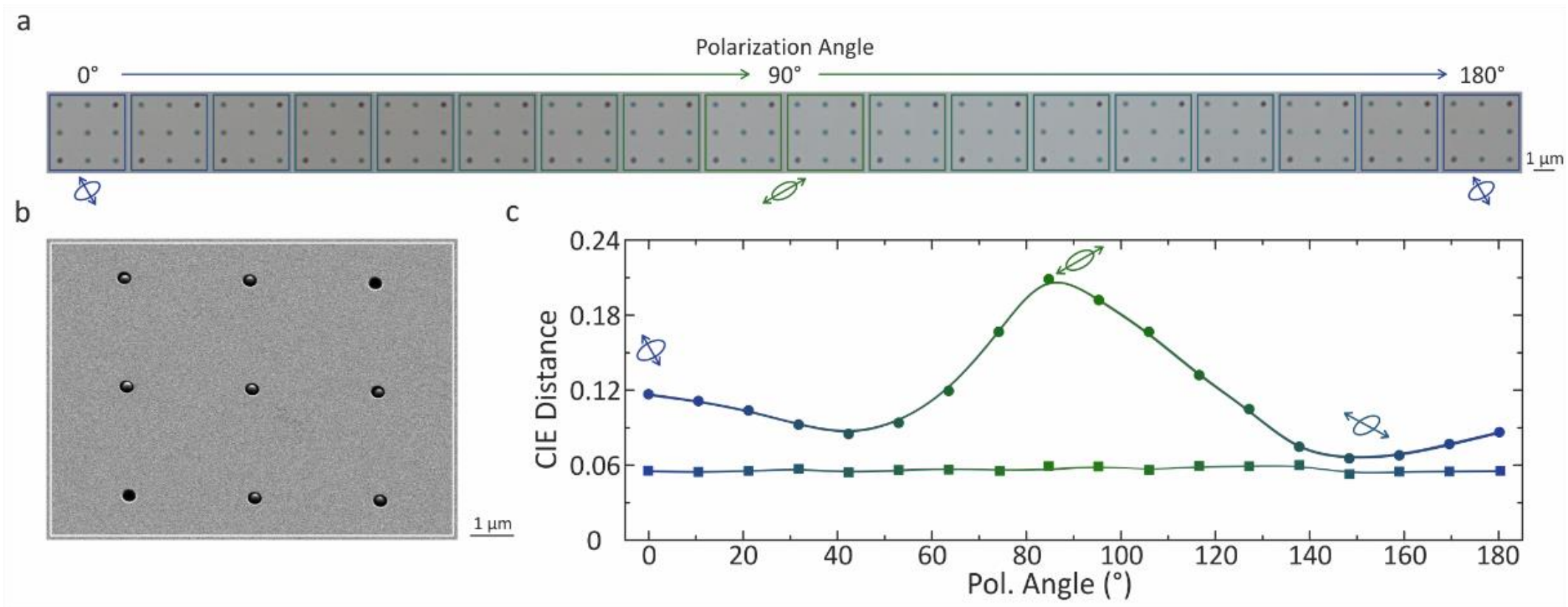

5) **Supplementary Information**

Caption of Supplementary Fig. SI15: Polarization sensitivity for filled round voids. a, Optical microscope images acquired for 18 different polarization states <u>ranging</u> from 0 ° to 180 °. **b**, SEM image of the corresponding field, indicating seven filled and two empty voids. **c**, CIE distance plotted as a function of the polarizer angle and compared

with Figure 5g. Only negligible changes in the CIE distance are observed for the ideally filled round voids.

6) **Experimental Results**

Advanced and polarization-dependent image analysis Moreover, in comparison to Figure 5g, the polarization sensitivity of the CIE distance for ideally filled round voids is presented in Supplementary Fig. 15. Here, the CIE distance does not show significant changes as a function of the polarizer angle.

Reviewer 2, Comment #1

> *"The paper reports an optical sensor for detecting plastic nanoparticles, that can differentiate between nanoplastics of different materials (PMMA, PET, PS), size, and shapes (spherical vs. elliptical shapes with different aspect ratios). The sensor is based on the concept of Mie void resonators, which are air void structures carved into a high-index GaAs substrate, that support optical resonances. The voids serve both as trapping sites for the plastic nanoparticles as well as optical readouts. The presence of nanoparticles alters the optical resonances of the Mie voids, leading to a change in color in bright-field imaging. The work provides comprehensive and convincing experimental evidence for the sensor's ability to differentiate between the size, shape, and material of plastic nanoparticles. In addition, the manuscript also leverages the polarization response of the void structures to increase the sensitivity of their sensor.*
> *Overall, I find the manuscript interesting, well-written and timely. Mie voids are a relatively new concept in nanophotonics, where new studies are emerging increasingly frequently. The proposed sensor leverages the trapping capability and the optical resonances of Mie voids, which I find innovative. For these reasons, I feel that publication in Nature Communications would be suitable. However, I have some comments that I would like addressed first."*

Our Response
We thank the reviewer for the positive feedback on the present manuscript and for suggesting its suitability for Nature Communications.

Reviewer 2, Comment #2

> *"It is clear that the voids are needed for the trapping of the particles. However, it is not obvious if the voids are needed for the optical read-out. Here, I would like to see optical control experiments, where the plastic nanoparticles are placed on e.g. flat GaAs, to clearly demonstrate that these nanoparticles cannot be differentiated without the Mie void resonances. Otherwise, if the Mie void resonances do not provide any optical functionality, then one might as well use a low-index substrate for trapping (i.e., glass) and dispel with the Mie void concept."*

Our Response
We thank the reviewer for this comment and for the suggestion to perform optical control experiments on flat GaAs substrates. These control experiments have been performed systematically to further clarify the necessity of the optical read-out approach by stepwise increasing the complexity of the deposited samples. Optical read-out of voids in high-index materials is required when more detailed information on the particle material or on precise particle dimensions is needed.

In a first experiment, PS particles with two different nominal diameters of 300 nm and 500 nm were simultaneously deposited onto the substrate. In the corresponding Supplementary Fig. 6, optical microscope images (a) acquired using a 100x objective are compared with the associated SEM images (b). From the optical microscope images, it is possible to identify two different sizes, yet no value can be given. However, due to the diffraction-limited resolution of the microscope and the minute size of the particles, more precise information, such as accurate size relations or interparticle distances, is lost. Extracting reliable information becomes even less feasible as the particles decrease in size. Consequently, this approach only allows for determining whether particles are present on the substrate or not.
Moreover, subsequent cleaning of the substrate using the standard procedure with water and ultrasonication is no longer possible, as this process would remove all deposited particles from the surface. Such cleaning processes, however, are usually necessary to avoid particles from forming clusters.
Extending the experiment toward more advanced tasks, such as distinguishing particles composed of different materials, further highlights the importance of the refractive-index-dependent optical response of the Mie voids. In Supplementary Fig. 7, a 1:1 mixture of PS and PMMA particles was

simultaneously deposited onto the substrate. In this case, distinguishing the different particle materials based solely on conventional optical microscopy becomes impossible, making the optical response of the void structures essential for reliable identification.
In a final control experiment, spherical and elliptical particles were simultaneously deposited onto a substrate. In this case, no clear distinction between spherical particles and elliptical particles with an aspect ratio of 2:1 can be made from the optical microscope image shown in Supplementary Fig. 8. To verify the observation, SEM images of the corresponding areas are presented in Supplementary Fig. 8b.
Overall, these experiments demonstrate that a flat GaAs substrate alone is insufficient for detailed particle characterization. While the platform allows for the detection of deposited particles and provides some general information regarding size differences, it fails when more specific applications or more detailed information about the sample are required. The void structures are essential when accurate particle size determination and precise differentiation between particles of varying dimensions or shapes are desired. Moreover, the optical resonances of the Mie voids become particularly important when probing effective refractive index variations, such as those present for different materials.
In principle, particle trapping should remain possible on a low-index substrate such as glass. Accordingly, particles can generally be sorted by size and shape, and empty voids should still be distinguishable from filled voids down to particle sizes of a few hundred nanometers. However, when discrimination relies on an effective refractive-index contrast, as is required, for example, for material identification, the low-index approach is no longer suitable because it lacks sufficient refractive-index sensitivity.

Action Taken:
To further clarify the importance of the Mie void structures for precise nanoplastic detection, three additional Supplementary Figures were added, and the Experimental Results section has been revised accordingly to introduce and discuss the new data. Furthermore, the numbering of all existing Supplementary Figures has been updated consistently throughout both the Supplementary Information and the manuscript.

1) **Supplementary Information**

Supplementary Fig. SI6: PS particles with different diameters deposited on a flat GaAs substrate. a, Optical microscope image of PS particles with nominal diameters of 300 nm and 500 nm simultaneously deposited on a flat GaAs substrate without fabricated Mie voids. Bottom, magnified view of the area highlighted by the black box. **b**, SEM image of the same area for comparison of the particle sizes. In the optical microscope image, particle size differences and interparticle distances can only be estimated approximately.

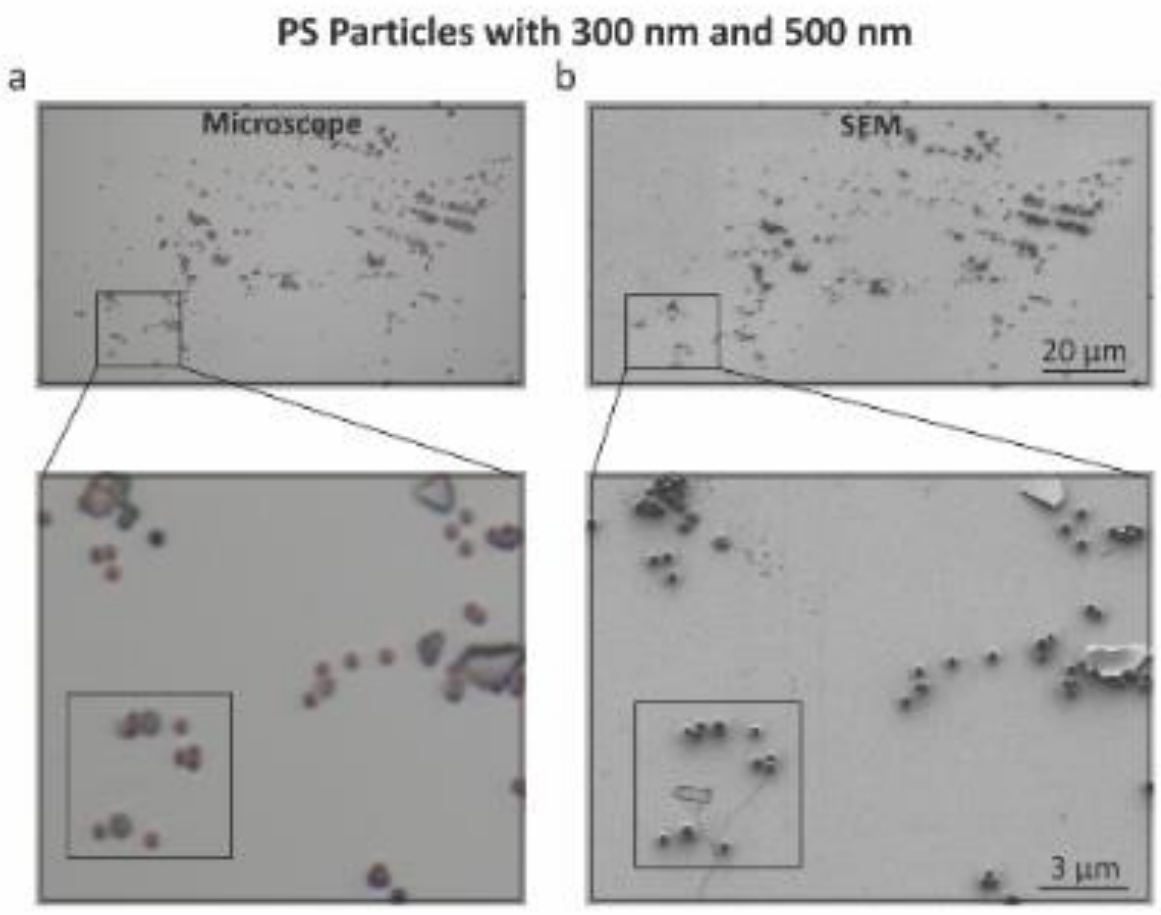

2) **Supplementary Information**

Supplementary Fig. SI7: Comparison of PS and PMMA particles on a flat GaAs substrate. a, Optical microscope image of PS and PMMA particles simultaneously

deposited on a GaAs substrate without fabricated Mie voids. A clear distinction between the different particle types is not possible. **b**, Magnified view of the highlighted region supporting this observation.

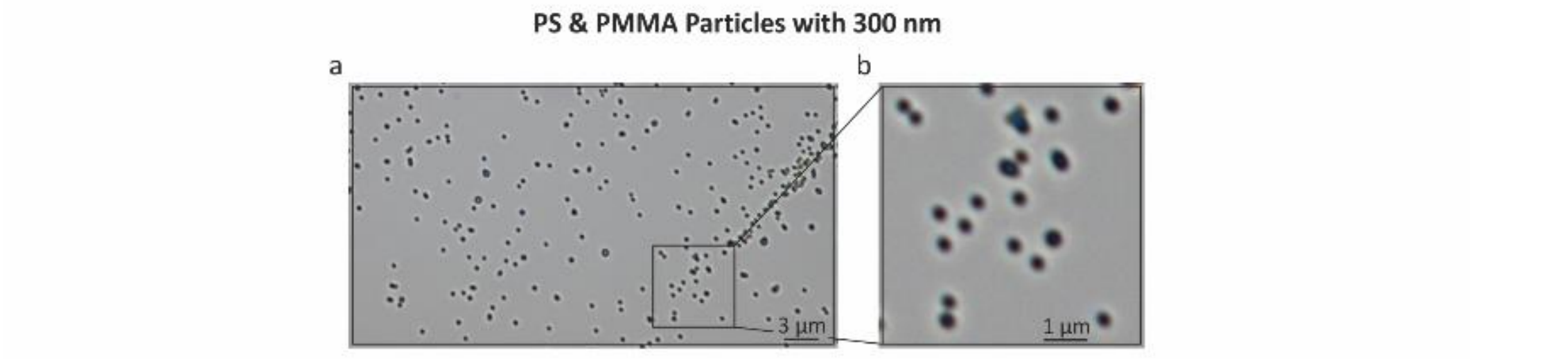

3) **Experimental Results**
   Material-sensitive detection Moreover, optical control experiments conducted on flat GaAs substrates without voids are presented in Supplementary Figs. 6 and 7. Here, PS particles with different nominal diameters (300 nm and 500 nm) and a mixture of PS and PMMA particles with nominal diameters of 300 nm are deposited. Analysis of the optical microscope images reveals that the refractive-index-dependent optical response of the Mie voids is crucial for enabling precise nanoplastic detection.
4) **Supplementary Information**
   Supplementary Fig. SI8: Investigation of spherical and elliptical particles on a flat GaAs substrate. a, Optical microscope image of spherical and elliptical PS particles simultaneously deposited on a flat GaAs substrate without fabricated Mie void structures. The inset depicts a magnified view of the highlighted region, where no clear distinction between spherical and elliptical particles can be observed. **b**, SEM image of the same region confirming this observation. In the small inset, the black marked area contains a spherical and an elliptical particle in close proximity.

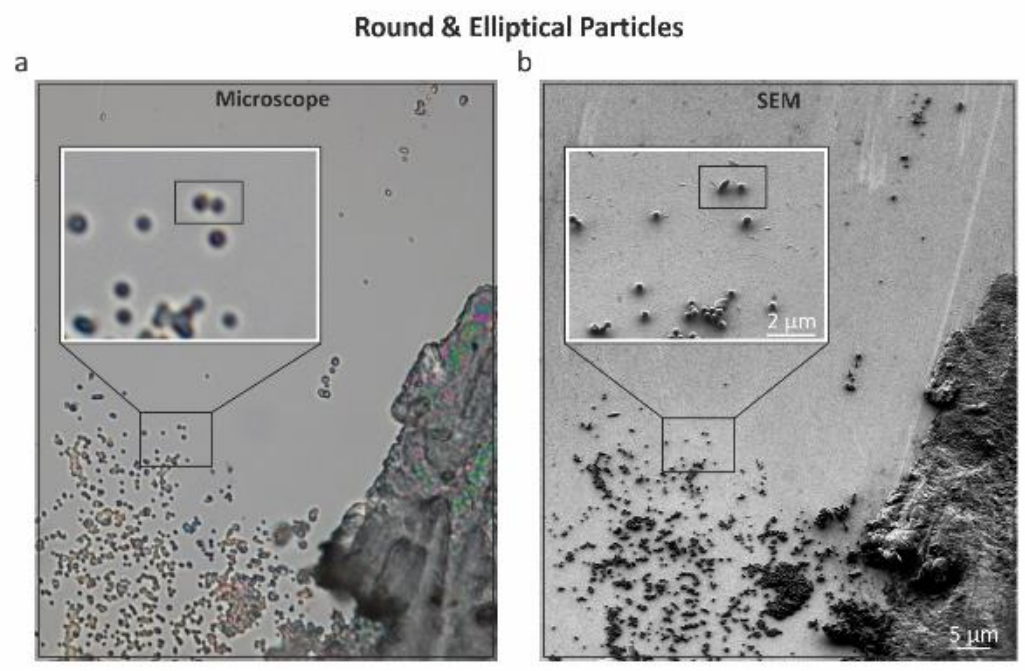

5) **Experimental Results**
   Shape-sensitive detection As before, an optical control experiment on a flat GaAs substrate, displayed in Supplementary Fig. SI8, is performed to further clarify the importance of the void structures. Particles with different shapes cannot be reliably distinguished using a bare substrate without fabricated voids.
6) **Supplementary Information**
   Corresponding adjustments of all Supplementary Figure numbers were made in both the Supplementary Information and the manuscript.

<u>**Reviewer 2, Comment #3**</u>

“Assuming that the Mie voids are critical for optical readout, then another point that I missed in the paper are design rules. With that I mean: how are the dimensions of the voids chosen? I suppose there are design considerations for the trapping as well as for the Mie void resonances. The manuscript would benefit from being explicit on how the dimensions

of the voids are chosen (and also explicitly stating these dimensions in the paper, i.e., void diameter, depth, pitch).”

Our Response
We thank the reviewer for this important question. Indeed, the detection system does not need to be optimized with respect to its optical response. Instead, the void geometries, including the diameter, the axis lengths of elliptical voids, and the depth, were solely designed to match the expected particle dimensions in the deposited sample. No explicit optimization of the void geometries to the optical resonances was performed. Since the readout is performed using an optical microscope, the full visible spectral range is available for probing. Owing to the selected void dimensions, optical resonances are therefore present within this spectral range, further underpinning the versatility of our concept.
In principle, however, the void geometries could be further optimized with respect to both the expected particle dimensions and the optical resonance properties. Such simultaneous optimization could further improve the detection fidelity, but was not required for the present experiments and may be the focus of future research.

Action Taken:
To clarify the design rules for the Mie void structures, we have added corresponding paragraphs to the Experimental Results section and to the Conclusion. Moreover, for all relevant figures (namely Figures 2-7), a short description was added with the specific void geometries, including diameter, major and minor axis lengths, depth, and pitch. These sections now read as follows:

1) **Experimental Results**
   General detection mechanism Please note that the voids used in the experiments were not optimized for specific optical resonances but were instead designed to match the expected particle dimensions in the specimen. The usage of broadband white-light microscopy then ensures the presence of optical resonances.
2) **Experimental Results**
   Material-sensitive detection The voids utilized in this experiment have a diameter of approximately 410 nm, a depth of 310 nm, and a center-to-center distance of 1.2 µm.
3) **Experimental Results**
   Shape-sensitive detection The circular voids possess a diameter of about 400 nm, while the elliptical voids have major and minor axis lengths of approximately 600 nm and 300 nm. The void depth is approximately 250 nm, and the center-to-center distance between adjacent voids is 1 µm.
4) **Experimental Results**
   Shape-sensitive detection The voids designed for particles with an AR of 2:1 have major and minor axis lengths of 620 nm and 340 nm, respectively. For AR = 3:1, the major and minor axis lengths are 800 nm and 310 nm, respectively. For an AR of 4:1, the corresponding axis lengths are 960 nm and 290 nm. The depth of the voids is approximately 320 nm, and the center-to-center distance increased from 1.2 µm to 1.5 µm and 2.0 µm.
5) **Experimental Results**
   Advanced and polarization-dependent image analysis The voids designed for particles with an AR of 2:1 possess a major axis length of approximately 610 nm, a minor axis length of 300 nm, a depth of about 350 nm, and a center-to-center distance of 1.2 µm.
6) **Experimental Results**
   Simultaneous morphology- and material-sensitive nanoplastic detection The circular voids have a diameter of 330 nm, whereas the elliptical voids possess major and minor axis lengths of 610 nm and 260 nm, respectively. The depth of the voids is approximately 280 nm, and the center-to-center distance is 1.2 µm.
7) **Conclusion**
   Future research may focus on the simultaneous optimization of void geometries to both match the deposited particles and enhance the optical resonances. In addition, surface functionalization could enable operation in more complex environments and further improve the device. Finally, the system performance could be validated using real environmental and biological samples, such as blood specimens.

__**Reviewer 2, Comment #4**__

"This is a subjective opinion, but to me, the title is not very clear. Especially the first part "size, shape, and material matter:" is bit too general. I think it could be valuable to include "Mie voids" in the title to elucidate the physical mechanism of the sensor, but I leave this decision to the authors."

Our Response
We thank the reviewer for this comment regarding the manuscript title and really appreciate that the reviewer leaves this decision to the authors.
Nevertheless, we take this suggestion seriously and agree that improving the clarity of the title could be crucial while trying to preserve the intended stylistic character. Therefore, we decided to revise the manuscript title to: "**Size, Shape, and Material matter: All-optical Mie void sensor for complex nanoplastic mixtures**".

Action Taken:
We have adjusted the title in the manuscript and the Supplementary Information accordingly.

1) **Manuscript Title**
   Size, Shape, and Material matter: All-optical Mie void sensor for complex nanoplastic mixtures.

__**Reviewer 2, Comment #5**__

Comment #5:
"On page 3, in the first two paragraphs of the result section, there is a short description on the trapping mechanism. In particular, the sentence on lines 106-108 prompted a few questions to me. How close do the particles have to be in size to the void dimensions? What happens if the particles are much smaller than the void dimensions, and what does "preferentially" mean in terms of success rate? I'm also wondering: Can you get a false negative result, i.e., particles that are present in the solution do not get trapped in the void and thus are not registered?"

Our Response
We thank the reviewer for these important questions, which indeed deserve a more detailed clarification. Overall, the trapping efficiency strongly depends on the degree of matching between the particle size and the void dimensions. The closer this match, the higher the trapping efficiency becomes. We have revised the Experimental Results section to clarify the trapping mechanism.

More specifically, when particles fit closely into the voids, the van der Waals interactions are maximized, allowing the particles to remain trapped even after extensive cleaning procedures. As the mismatch between particle and void size increases, these interaction forces decrease. If the voids are substantially larger than the particles, the particles interact with only a fraction of the surrounding surfaces. Consequently, the total interaction area is reduced, resulting in weaker van der Waals forces and making particle removal during cleaning more likely.
In contrast, when the voids are too small, particles cannot enter them and instead rest on top of the structures, where they can be readily removed during subsequent washing. Defining an exact threshold for the acceptable size mismatch is difficult, as it depends on the specific particle and void dimensions used for each experiment.
Nevertheless, our previous experiments published in Nature Photonics **19**, 1138-1145 (2025) indicate that for spherical particles with a nominal diameter of 350 nm, a size offset of approximately 80 nm in both directions can still allow particle trapping. For such mismatched voids, filling probabilities of around 5 - 10 % were still observed. The term "preferentially" was thus intentionally chosen in this context, as the trapping process is not binary. Slight deviations in particle-void matching do not

eliminate trapping entirely. Rather, such deviations reduce the van der Waals forces and consequently lower trapping efficiencies while still allowing a sufficient fraction of particles to remain trapped even after cleaning.
Additionally, it should be noted that the particles themselves exhibit an intrinsic size distribution. A nominal diameter of 300 nm does not imply a perfectly monodisperse population. Dynamic Light Scattering measurements reveal a relatively broad particle size distribution, as indicated in Supplementary Fig. SI2. This diameter variation influences the matching conditions between particles and voids and therefore affects the trapping efficiency.
A false-negative outcome is, in principle, possible, as particle trapping is a probabilistic process. Statistical fluctuations can potentially lead to either over- or underrepresentation of trapped particles, causing the experimentally observed trapping efficiency to deviate from the true particle-void matching behavior. Therefore, reliable evaluation requires either a sufficiently large number of voids to achieve meaningful statistical significance or multiple independent samples to generate averaged results and reduce the probability of false-negative results.

Action Taken:
We have added two paragraphs to the Experimental Results and Methods sections to further clarify the trapping process and efficiency.

1) **Experimental Results**
   General detection mechanism When the void dimensions and particle sizes deviate from each other, the interaction forces are reduced, increasing the likelihood of particle removal during subsequent cleaning steps (see Methods). Previous experiments, however, have shown that for particles with a nominal diameter of 350 nm, a diameter mismatch of approximately ±80 nm can still result in trapping efficiencies of around 5 - 10 % [49]. To maximize trapping efficiencies, the void dimensions are therefore designed to closely match the particle size.
2) **Methods**
   Nanoplastic Particles During this cleaning procedure, mismatched particles are removed when the interaction forces are insufficient to retain them within the voids. Consequently, this cleaning acts as a filtering step that translates the degree of matching between particle and void dimensions into the observed trapping efficiency. This relationship enables the precise determination of particle size and shape using Mie voids.

<u>**Reviewer 2, Comment #6**</u>

> *“Along the same lines as point 4, will fabrication imperfections of the void structure impact the sensor capabilities and to what extent?”*

Our Response
We thank the reviewer for raising this important point. Fabrication imperfections of the voids can indeed affect the sensing performance by influencing the trapping efficiency. However, the employed fabrication process, based on EBL combined with dry etching of semiconductor materials (GaAs), is highly established and provides very good dimensional control. As a result, fabrication-induced deviations are sufficiently small and enable the reliable production of void arrays with high accuracy.
Importantly, these fabrication tolerances are comparatively small when contrasted with the particle size distributions shown in Supplementary Fig. SI2. The DLS measurements reveal relatively broad particle size distributions with FWHM values of 389 nm (PS), 293 nm (PMMA), and 225 nm (PET). Consequently, the inherent variation in particle dimensions is expected to have a substantially greater impact on trapping behavior than fabrication-related deviations of the void structures.

Action Taken:
To clarify the impact of the fabrication imperfections, a sentence was added to the Experimental Results section. It now reads as follows:

1) **Experimental Result**

Material-sensitive detection These determined size variations for particles with nominal diameters of 300 nm indicate that intrinsic differences in the particle dimensions dominate over other sources of uncertainty, such as fabrication-induced inaccuracies of the sensor structures.

Reviewer 2, Comment #7

"In relation to SI Figures 5 and 7, the authors investigate cases where unwanted particles are trapped in the voids. Without SEM imaging, how can the sensor differentiate between if such an unwanted particle is from a dirt particle or an elliptical particle tip-first?"

Our Response

We thank the reviewer for this valuable comment and interesting question. In general, SEM imaging is not required if the goal is only to determine whether the color of a void corresponds to a specific void state, for example, filled or empty. If a void is filled with debris, for instance, it exhibits a clearly different color and can therefore be distinguished from the expected void states. SEM imaging is only required when more detailed information about a specific void state is needed.

In our case, SEM characterization is necessary because we aim to unambiguously assign each observed color to a specific void state. This allows us, for example, to identify cases in which unusual colors arise from non-typical configurations.
If SEM imaging is not available, unambiguous identification becomes more challenging. For example, in Supplementary Fig. SI5, the individual spot shown in Fig. SI5c could not be conclusively identified based on the optical measurement alone. It could only be determined that this spot does not belong to any of the three distinct color groups observed in the measurements, without providing further information on its physical origin.

Action Taken:

We have revised two passages in the Experimental Results sections to improve clarity regarding how unwanted void states can be distinguished from correctly filled or empty voids.

1) **Experimental Results**

   Shape-sensitive detection Such incorrectly filled voids can therefore be readily distinguished from empty or correctly filled voids solely based on their resonance color following initial verification by SEM imaging.

2) **Experimental Results**

   Shape-sensitive detection Consequently, the resulting optical response, and thus the observed color, remains distinct, enabling unambiguous differentiation between spherical and ellipsoidal particles in circular voids after initial reference measurements.

Reviewer 2, Comment #8

"In SI Figure 10, simulations of the void structure with spherical plastic nanoparticles are presented. Why are the simulated colors different from the experimental ones?"

Our Response

We thank the reviewer for this important comment. In simulating our sample, we aimed to develop a model that makes as few assumptions as possible while still reliably capturing the behavior of our system. Consequently, several experimental details, such as for example the inclined sidewalls of the fabricated voids, were not included in order to keep the model complexity to a minimum. As a result, certain differences between the simulated and experimentally observed void colors are expected.

In more detail, we indeed observe that the simulated colors are generally positioned further toward the outer regions of the CIE diagram and thus closer to pure red, green, and blue colors. Consequently, these data points usually exhibit a broader distribution compared with the

experimentally obtained data, and the simulated color responses appear more pronounced than those observed experimentally.
However, these differences are expected and arise because the simulations are not optimized to fully account for all experimental factors. For example, characteristics of the imaging system, as well as geometric deviations introduced during sample fabrication are not included in the simulation model. In detail, the simulations assume idealized structures and do not include fabrication-induced variations of the void geometry or deposited particles, both of which can significantly influence the optical response. In particular, the void depth, which is estimated from SEM measurements, cannot be determined with perfect accuracy and has a substantial impact on the resulting simulated color.
Furthermore, no brightness correction is applied in the simulations, and the white balance is referenced to an ideal white color. In contrast, the experimental images are white-balanced using the reflection signal from the bare GaAs substrate. These differences in image processing directly influence the resulting color representation and can therefore contribute to deviations between simulation and experiment.
Another important aspect concerns the PET particles used in the experiments. These particles consist of at least 75 % PET, while the remaining fraction comprises a PS core required for particle fabrication. This composite structure influences the effective refractive index of the particles. However, as the exact composition and internal structure are not fully known, the refractive index of pure PET was used in the simulations. This simplification represents an additional source of deviation between simulated and experimental results.
Overall, the agreement between simulations and experiments could potentially be improved by introducing additional correction factors related to contrast, brightness, and imaging conditions and by increasing the simulation complexity, which would alter the resulting color distribution and position within the CIE diagram. However, the primary objective of the simulations was not to achieve exact color reproduction, but instead, the simulations were intended to provide a general prediction of whether refractive index differences could be distinguished and to support the interpretation of the experimentally established reference measurements used for material identification.

Action Taken:
We have added a section to the Experimental Results to explain the differences between the experimental observations and the simulation results. Moreover, we have revised the Methods section related to the simulations to improve the understanding and description of the simulation procedure.

1) **Experimental Results**
   Simultaneous morphology- and material-sensitive nanoplastic detection This assignment is supported by simulated reflection spectra and corresponding colors for the three materials and the empty case (Supplementary Fig. 16). Please note that deviations between the simulated and experimentally observed colors can arise due to experimental factors such as variations in void geometries, particle size distributions, effective refractive indices, and differences in the applied white balance. Moreover, reference measurements were performed with only a single particle type for PS (Supplementary Fig. 17), PMMA (Supplementary Fig. 18), and PET (Supplementary Fig. 19).
2) **Methods**
   Simulations Please note that, due to fabrication constraints, the PET particles consist of at least 75 % PET, while the remaining fraction is formed by a PS core. This composite structure influences the effective refractive index of the particles. However, since the exact composition is not known, this effect is not explicitly considered in the simulations.
3) **Methods**
   Simulations In contrast to the experimental procedure, where the white balance is calibrated using the reflection of the bare GaAs substrate, the simulations use an ideal white reference for the balancing.
4) **Methods**
   Simulations An improved analytical model for predicting the optical color response was recently published [58].
5) **References**

58. Schwab, J. et al. Illuminating Mie Voids: An Analytical Model for Nanophotonics. ACS Photonics (in press, 2026).

Reviewer 2, Comment #9

“In SI Figure 11-13, a second set of reference measurements (i.e., pure voids without nanoplastic) are presented. Why do you need a second set of reference measurements? Are the sensors different?”

Our Response
We thank the reviewer for this comment. In principle, a second reference set is not inherently required.
However, since the samples investigated in Figure 7 were fabricated in a separate fabrication run, in contrast to the sample in Figure 2, there might be a chance for slight sample variations. To ensure that the reference measurements fit the experimental observations perfectly, a second set of reference measurements was created. Therefore, performing a second reference measurement improves calibration accuracy and enhances the reliability of the material identification process. For samples fabricated within the same process run, an additional reference set would generally not be required.

Action Taken:
We have added a sentence to the Experimental Results to explain why a second set of reference measurements was used.

1) **Experimental Results**

   Simultaneous morphology- and material-sensitive nanoplastic detection A second set of reference measurements was employed to ensure the highest reliability of the material discrimination.

Reviewer 3, Comment #1

> *"This manuscript demonstrated that Mie voids can selectively separate nanoplastics with different sizes and shapes by capturing them in the voids with different shapes and detect difference in materials constructing the nanoplastics by simply observing the change in resonant color of the Mie voids filled by them with reflection optical microscope. Issue caused by nanoplastics is currently a subject of major environmental concern, which potentially impacts on lives strongly. The authors previously provided a simple and smart approach based on nanophotonics using isotropic circle-shaped Mie voids to address the issue that convenient analytical methods for nanoplastics still lack (D. Ludescher et al., Nat. Photon., 19, 1138. (2025)). The current manuscript presents the advancement from the previous work. The manuscript demonstrated the selective detection of nanoplastics with different anisotropy using Mie voids with different anisotropy, the distinction of nanoplastics composed of different materials and the advanced optical detection from the color difference under orthogonal linearly polarized light illumination to improve sensitivity. Although the basic concept of the research, optical sieve for nanoplastic detection, is the same as the author's previous work, I believe that this study is still worthy of being published in Nature communication and deserves to be widely addressed because that the current study emphasized versatility and new functionality applicable to real nanoplastics present in nature. In addition, the application of Mie voids evokes potential impacts on wide variety of scientific fields as localized surface plasmon resonance of metal nanoparticles has done."*

Our Response

We thank the reviewer for their positive feedback on our manuscript. We appreciate the reviewer's recognition of the versatility and novel functionality enabled by the presented work for the nanoplastic detection method.

Reviewer 3, Comment #2

> *"Coordination x and y should be italic."*

Our Response:

We thank the reviewer for this careful comment. We have revised the corresponding Figures in the Experimental Results section and Supplementary Information by formatting the axis labeling *x* and *y* in italic font. To maintain a consistent style throughout the manuscript, we also revised Figure 3 by formatting *R*, *G*, and *B* in italic font.

Action Taken:

1) **Experimental Results**
   The axis labels in the CIE color diagrams in Figures 2, 5, and 7 were corrected to an italic font.
2) **Experimental Results**
   The axis labels in the RGB color diagram in Fig. 3 were corrected to an italic font.
3) **Supplementary Materials**
   The axis labels in the CIE and RGB color diagrams in Fig. SI3, Fig. SI4, Fig. SI5, Fig. SI9, Fig. SI17, Fig. SI18, and Fig. SI19 were corrected to an italic font.

<u>**Reviewer 3, Comment #3**</u>

> *"Figure 3 caption: Regarding the description "**a & b**, Optical microscope and **c & d**, SEM images of the detection strip with elliptical (left rows) and round (right rows) voids." I believe that optical microscope images are a and c not a and b. Also I believe that SEM images are b and d not c and d."*

Our Response:
We sincerely appreciate the reviewer's careful attention in noticing this mistake. We have revised Figure 3 by assigning the correct images. The corresponding text in the manuscript has also been revised accordingly.

Action Taken:
We have revised the Experimental Results section by adjusting Figure 3 and the corresponding text in the manuscript. It now reads as follows:

1) **Experimental Results**

   Correction of Figure 3 Figure 3 was adjusted such that the optical microscope images now correspond to Figs. 3a and 3b. The SEM images correspond to Figs. 3c and 3d.

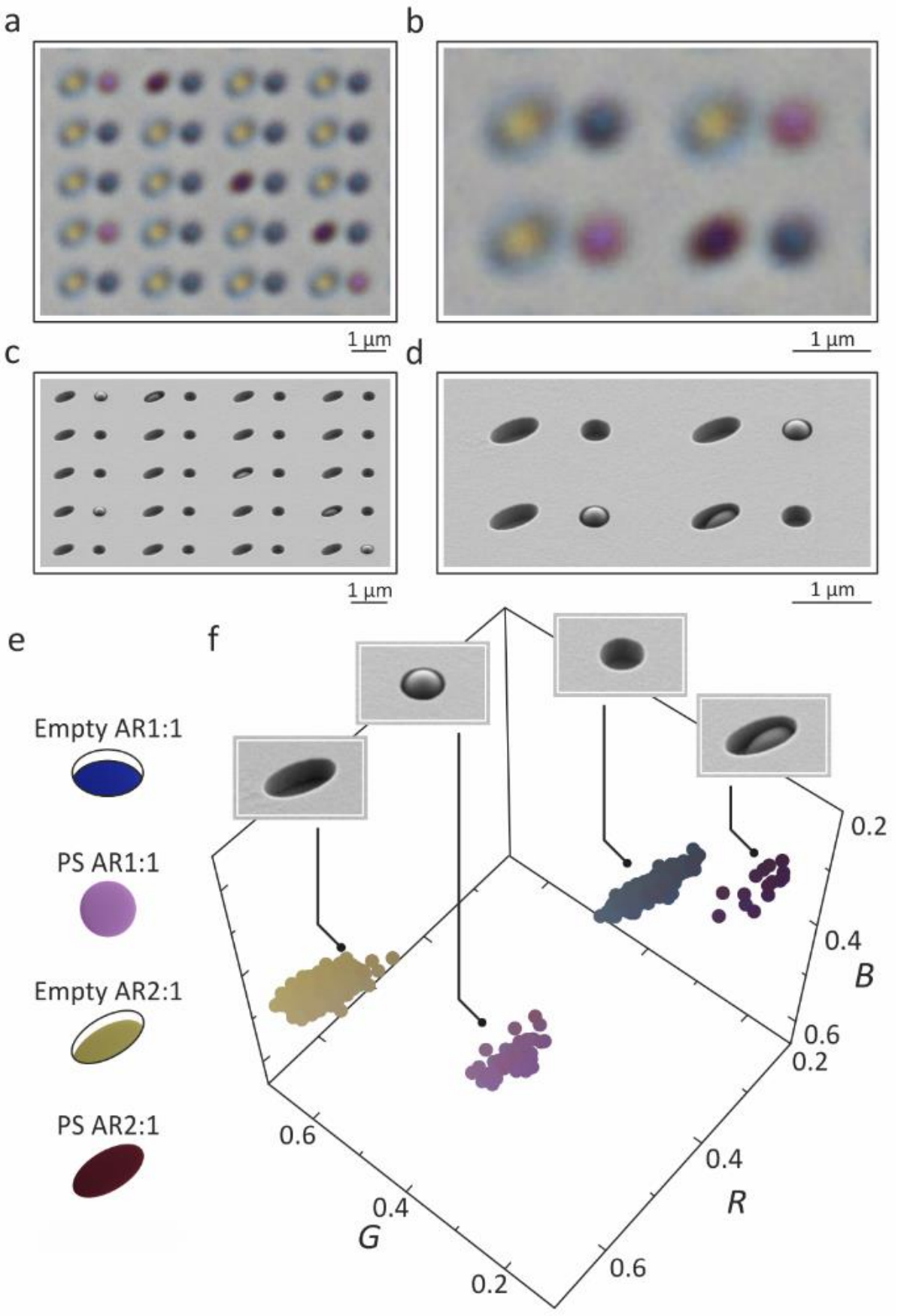

2) **Experimental Results**

   Shape-sensitive detection Optical microscope images of the detection strip are presented in Figure 3a and Figure 3b, displaying in periodic arrangement elongated voids (left) and round voids (right), respectively. Here, linear polarization in the illumination path of the optical microscope along the short axis of the voids is used to increase the image contrast. The corresponding SEM images are presented in Figure 3c and Figure 3d.

Reviewer 3, Comment #4

> *"Conclusion: Line 312: Regarding the description "we introduce a purely optical platform that enables the simultaneous, shape…" I believe that shape detection is not purely optical."*

Our Response:
We thank the reviewer for this comment and agree that the phrase "purely optical" may be misleading in this context, as only the detection is performed optically, whereas the sorting and trapping of the particles are not optical processes. We have therefore removed the word "purely" and revised the sentence to improve the clarity of the conclusion.

Action Taken:
The conclusion has been revised. It now reads as follows:

1) **Conclusion**
   In summary, we introduce a sensing platform that enables the simultaneous, shape- and material-sensitive detection of individual nanoplastic particles with diameters down to 300 nm, based on the optical response of Mie void resonators.

Reviewer 3, Comment #5

> *"Methods: Descriptions should be the past tense.*
> *Line 518: "Monodisperse PS spheres (350 nm diameter) are embed-ded in an polyvinylalcohol matrix" Why "-" is between "embed" and "ded"?*
> *Line 572: "n" of refractive index should be italic."*

Our Response
We thank the reviewer for the comment. We corrected the spelling mistake in line 518 by removing the unnecessary hyphen. Moreover, the refractive index symbol "n" was corrected to italic font in line 572.

Action Taken:
The two sections in the Methods now read as follows:

1) **Methods**
   Nanoplastic Particles Monodisperse PS spheres (350 nm diameter) are embedded in a polyvinylalcohol matrix (Mowiol. 8-88, MW = 67000, 86.7 – 88.7 % hydrolysis, Merck / Sigma-Aldrich) cast as thin films and subsequently mounted in a mechanical stretching device.
2) **Methods**
   Simulations The structures are modeled as conical voids etched into a GaAs substrate, with air (n = 1) occupying the void volume and the superstrate region.
